# Eosinophils promote pulmonary matrix destruction and emphysema via Cathepsin L

**DOI:** 10.1038/s41392-023-01634-x

**Published:** 2023-10-11

**Authors:** Xia Xu, Tao Yu, Lingling Dong, Rainer Glauben, Siyuan Wu, Ronghua Huang, Shiwei Qumu, Chenli Chang, Jing Guo, Lin Pan, Ting Yang, Xin Lin, Ke Huang, Zhihua Chen, Chen Wang

**Affiliations:** 1 Department of Immunology, School of Basic Medical Sciences, Capital Medical University, Beijing, China; 2 Department of Pulmonary and Critical Care Medicine, China-Japan Friendship Hospital, Beijing, China; 3 Key Laboratory of Respiratory Disease of Zhejiang Province, Department of Respiratory and Critical Care Medicine, Second Affiliated Hospital of Zhejiang University School of Medicine, Hangzhou, Zhejiang China; 4 Department of Gastroenterology, Infectious Diseases, and Rheumatology, Campus Benjamin Franklin, Charité-University Medicine Berlin, Berlin, Germany; 5 Institute of Respiratory Medicine, Chinese Academy of Medical Science, Beijing, China; 6 Institute of Clinical Medical Sciences, China-Japan Friendship Hospital, Beijing, China; 7 Institute for Immunology, Tsinghua University School of Medicine, Beijing, China

**Keywords:** Respiratory tract diseases, Inflammation

## Abstract

Patients with chronic obstructive pulmonary disease (COPD) who exhibit elevated blood eosinophil levels often experience worsened lung function and more severe emphysema. This implies the potential involvement of eosinophils in the development of emphysema. However, the precise mechanisms underlying the development of eosinophil-mediated emphysema remain unclear. In this study, we employed single-cell RNA sequencing to identify eosinophil subgroups in mouse models of asthma and emphysema, followed by functional analyses of these subgroups. Assessment of accumulated eosinophils unveiled distinct transcriptomes in the lungs of mice with elastase-induced emphysema and ovalbumin-induced asthma. Depletion of eosinophils through the use of anti-interleukin-5 antibodies ameliorated elastase-induced emphysema. A particularly noteworthy discovery is that eosinophil-derived cathepsin L contributed to the degradation of the extracellular matrix, thereby leading to emphysema in pulmonary tissue. Inhibition of cathepsin L resulted in a reduction of elastase-induced emphysema in a mouse model. Importantly, eosinophil levels correlated positively with serum cathepsin L levels, which were higher in emphysema patients than those without emphysema. Expression of cathepsin L in eosinophils demonstrated a direct association with lung emphysema in COPD patients. Collectively, these findings underscore the significant role of eosinophil-derived cathepsin L in extracellular matrix degradation and remodeling, and its relevance to emphysema in COPD patients. Consequently, targeting eosinophil-derived cathepsin L could potentially offer a therapeutic avenue for emphysema patients. Further investigations are warranted to explore therapeutic strategies targeting cathepsin L in emphysema patients.

## Introduction

Chronic obstructive pulmonary disease (COPD) is a varied disease with widespread prevalence globally.^1,2^ Emphysema and small airway disease encompass major features of COPD. Current therapies, including bronchial dilators and inhaled corticosteroids, primarily influence airway inflammation to relieve symptoms, reduce lung function decline, and inhibit acute exacerbation.^3^ However, pathological mechanisms responsible for COPD and emphysema development are incompletely understood. Consequently, apart from quitting smoking, there is currently no effective therapy to reverse this condition.

The prevailing theory concerning the emergence of emphysema revolves around an imbalance between proteases and antiproteases within the corresponding tissues. α1-Antitrypsin acts as a suppressor of neutrophil elastase, and its deficiency leads to an inadequately regulated release of elastase. Consequently, the lung’s connective tissue undergoes elastin degradation, a phenomenon primarily linked to the advancement of emphysema.^4^ Elastase-induced emphysema brings about histological and morphological traits akin to those observed in panacinar emphysema.^5^ Our exploration utilizing multiple models of elastase instillation demonstrated that repeated exposure to elastase significantly triggers lung eosinophilia and consequent emphysematous changes;^6^ nonetheless, the exact mechanisms and roles played by eosinophils in these processes remain to be fully illuminated.

Proteinases, including neutrophil elastase, matrix metalloproteases (MMPs), and cysteine proteases, participate in the development of emphysema as well as small airway remodeling in COPD. Genschmer et al.^7^ determined that elastase delivered by exosomes from activated neutrophils binds to the extracellular matrix (ECM) and degrades it, to promote emphysema. Many studies employing a cigarette-smoking mouse model of COPD suggest that enhanced MMP expression in macrophages and cathepsin S (CTSS) are essential for the development of emphysema in neutrophil-related COPD.^8–11^ COPD is classified as a neutrophilic airway disease. However, the role of eosinophils in its course has attracted recent attention. Results from extensive cohort studies have indicated that patients with COPD possessing blood eosinophil levels >200/mm^3^ have worsened lung function, more severe emphysema, and an elevated risk of exacerbations.^12,13^ An eosinophilic phenotype of COPD has been outlined, and patients with heightened blood eosinophil counts are recommended to receive inhaled corticosteroids.^14^

Therapies targeting Th2 or eosinophilic inflammation, such as interleukin (IL)-5 and IL-33/ST2, have beneficial effects in COPD patients. However, monoclonal antibodies targeting the IL-5 receptor do not decrease aggravations. This suggests that further study is warranted.^15–17^ The precise role of eosinophils in COPD pathophysiology is not adequately understood compared to other eosinophil-related disorders, such as asthma, while the exact role of eosinophils in asthma is being investigated.^18,19^ Our previous work outlined eosinophilic inflammation in porcine pancreatic elastase (PPE)-induced mouse models of emphysema.^6^ Another study has demonstrated that eosinophil-derived IL-13 promotes MMP-12 production in alveolar macrophages and emphysema development.^20^ However, it is unclear whether eosinophils participate in COPD development, such as ECM degradation through the secretion of proteinases.

This study identified a distinct eosinophil phenotype in emphysematous mouse lungs, revealing the critical role of eosinophil-derived cathepsin L (CTSL) in the development of emphysema. In addition, CTSL expression in eosinophils was positively correlated with emphysema in COPD patients. Therefore, CTSL is a potential therapeutic target in emphysema patients, especially those with eosinophilia.

## Results

### Eosinophils accumulate in elastase-induced emphysematous mouse lungs

**Fig. 1:**
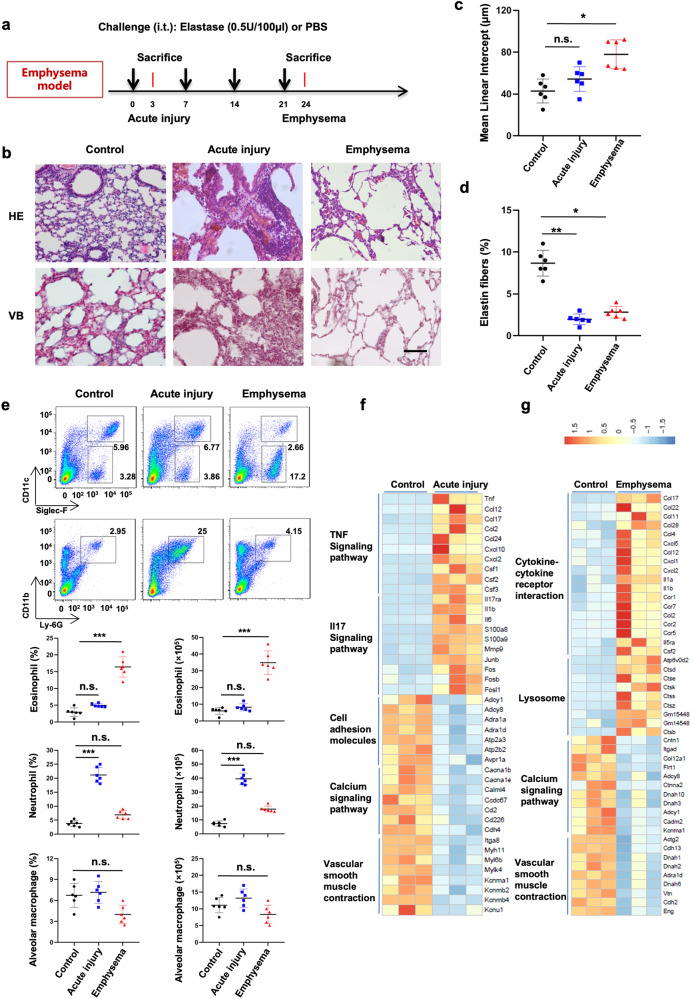
**Eosinophils accumulate in chronic elastase-induced pulmonary emphysema. a** Elastase (0.5 U/100 µL) was administered intranasally to C57BL/6 mice on a weekly basis for four consecutive weeks. Terminal analysis was carried out either 3 days following the initial challenge (acute injury model) or subsequent to the last challenge (emphysema model). **b** Pulmonary histological sections were subjected to hematoxylin and eosin (HE) and Victoria Blue (VB) staining for characterization (original magnification ×1000 for all panels, bars represent 100 μm). **c** Mean linear intercept, and **d** elastin fiber content across the treated mouse lungs. **e** Complete lung cells were isolated and analyzed for eosinophil (CD11c^-^SiglecF^+^), neutrophil (CD11b^+^Ly6G^+^), and alveolar macrophage (CD11c^+^SiglecF^+^) levels using flow cytometry. **f**, **g** Heatmap of clustered gene expression markers differentially expressed between control and acute injury groups **f** or between control and emphysema groups in lung tissues **g**. The data represent three independent experiments. Data are the mean±s.e.m. ^*^P < 0.05, ^**^P < 0.01 and ^***^P < 0.001 using a two-tailed unpaired *t*-test. In **a**–**e**, *n* = 6 mice per group

A mouse emphysema model was formed via weekly intratracheal instillation of PPE over the course of 4 weeks.^6^ Acute injury involved initial PPE instillation to examine PPE-induced early reactions in the lungs. The mice were sacrificed on day three after the acute injury (Fig. 1a). Severe emphysema was assessed in mouse lungs following the fourth treatment, and the average linear intercept was significantly increased in emphysema models compared to the controls (Fig. 1b, c). Elastic fibers were reduced in the lung alveolar walls in acute and emphysema models (Fig. 1d). Moreover, hematoxylin and eosin (H&E) staining of the respective lung tissues demonstrated that PPE triggered an inflammatory cell influx into the lungs following the first instillation (Fig. 1b), further analyzed using flow cytometry (Supplementary Fig. 1a). Neutrophils primarily accumulated in the acute injury model on day three following the first PPE instillation, while mice exhibited increased eosinophil numbers in emphysematous lungs after the fourth PPE treatment. Alveolar macrophage levels remained steady in lungs harboring acute injury or emphysema compared to the controls (Fig. 1e). These data indicated a switch from neutrophilic to eosinophilic inflammation upon repeated PPE challenges.

Following this, we compared the transcriptomes of lung cells across the three groups. Gene Ontology (GO) analysis revealed the presence of significantly upregulated genes in immune-related pathways (especially in TNF and IL-17 signaling within the acute injury group) compared to the control groups. Conversely, pathways involved in calcium signal transduction, vascular smooth muscle contraction, and cell adhesion were substantially downregulated (Fig. 1f and Supplementary Fig. 1b). The levels of inflammatory cytokines related to type 2 inflammation (including CCL11, CCL17, and CCL22) were significantly elevated in the emphysema model compared to the controls. Additionally, genes linked to lysosomal function were enriched in the emphysema models (Fig. 1g and Supplementary Fig. 1c). Similarly, gene set enrichment analysis showcased that the IL-17 signaling pathway was increased in the acute injury models. In contrast, genes linked to lysosomes were downregulated in the emphysema models (Supplementary Fig. 1d, e). These findings confirm the transition from acute neutrophilic inflammation to chronic eosinophilic inflammation in PPE-induced emphysema models.

### Characterization of lung cell subpopulations

**Fig. 2:**
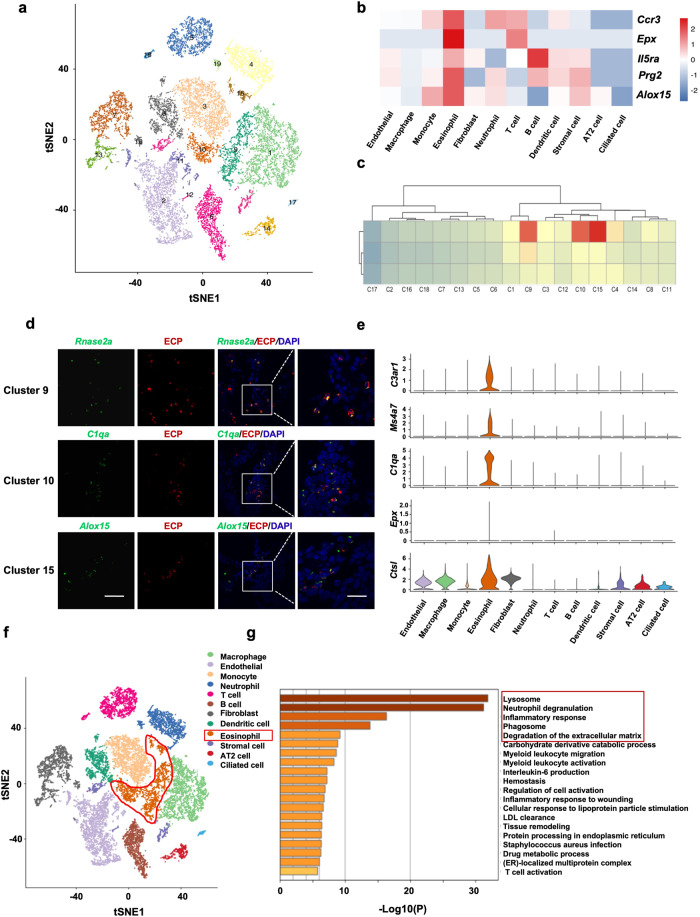
**Characterization of lung cell subpopulations**. Mice were randomly divided into four groups to identify cell types in lung tissues: asthma models, PPE-emphysema models, PPE-recovery models, and control groups (*n* = 6 for each group). scRNA-seq analysis of entire lung cells isolated from one mouse for each group. **a** tSNE plot of single cells colored based on primary cell clusters. **b** Heatmap of differentially expressed marker genes across cell types. **c** Correlation coefficient analysis of the shared cell types between single-cell RNA sequencing and bulk RNA sequencing of eosinophils. **d** Immunofluorescence staining for ECP (red), in situ hybridization for *Rnase2a*, *C1qa*, and *Alox15* mRNA (green), and 4′,6-diamidino-2-phenylindole (DAPI, blue) in emphysema mouse lung tissue samples. The scale bars for low and high magnification are 50 μm and 20 μm, respectively. Images represent 6 emphysema samples. **e** Violin plots illustrate the expression of selected markers in eosinophils. **f** The t-SNE plot outlines eosinophil cell types in lung tissues. **g** Enriched Gene Ontology (GO) functions of upregulated genes in eosinophils

Single-cell RNA sequencing (scRNA-seq) was performed on protease-dissociated cells from mouse lungs to investigate if eosinophils participate in emphysema development. An ovalbumin-induced asthma model was constructed to compare the functioning of eosinophils in asthma and eosinophilic COPD to uncover the full spectrum of eosinophil differentiation and discover potential eosinophil populations. Emphysema recovery models were engineered to determine the primary factors influencing recovery from eosinophilic emphysema (Supplementary Fig. 2a). High-dose instillation primarily induced neutrophilic inflammation on day 3. Macrophage accumulation on day 10 was linked to the development of emphysema, and inflammation was no longer detected after 2 weeks (Supplementary Fig. 2b, c). Lung tissues were dissociated into single-cell suspensions for transcriptome analysis of immune and stromal cells of the lungs.^21^ A total of 19,775 high-quality cells containing 11,405 genes were separated into 19 clusters after quality control using a t-distributed stochastic neighbor embedding method (Fig. 2a and Supplementary Fig. 2d). Cluster-specific genes were employed to annotate cell types based on classical markers described in previous studies: T cells (CD3e^+^), B cells *(*Cd79a^+^), monocytes (*Ly6c2*^+^), dendritic cells (Cd209a^+^), macrophages (*Cd200r*^*+*^), fibroblasts (*Col1a1*^+^), and endothelial cells (*Cdh5*^+^). Cluster 19 represented granulocyte cell and erythrocytic markers, considered to be doublets (Supplementary Fig. 2e).^22,23^ Clusters 9, 10, and 15 highly expressed classical eosinophil markers, such as *Ccr3*, *IL5ra*, *Epx*, *Prg2*, and *Alox15* (Fig. 2b). We compared the scRNA-seq-defined eosinophil populations to classically defined eosinophils for an improved characterization of eosinophils in mouse lungs isolated via fluorescence-assisted cell sorting (FACS) based on CD11c and SiglecF expression^24^ from the lungs of the experimental models and analyzed them through RNA-seq (Supplementary Fig. 2f, g). Regression-based deconvolution analysis demonstrated a strong relationship between the expression patterns of clusters 9, 10, and 15 and classically defined eosinophils (Fig. 2c). Clusters 9, 10, and 15 were the major representatives of *Rnase2a*, *C1qa*, and *Alox15* expression. In situ hybridization of *Rnase2a*, *C1qa*, and *Alox15* mRNA and immunostaining for ECP uncovered that clusters 9, 10, and 15 were distributed separately throughout a subset of ECP^+^ cells (Fig. 2d). Clusters 9, 10, and 15 (expressing *C3ar1*, *Epx*, *Ms4a7*, *C1qa*, and *Ctsl*) were recognized as eosinophils (Fig. 2e, f). GO enrichment analysis of each cluster demonstrated that eosinophils were associated with lysosomes, degranulation, inflammatory responses, phagosomes, and ECM degradation (Fig. 2g). This indicates that eosinophils are critical for the development of emphysema.

### Characterization of distinct eosinophil states

**Fig. 3:**
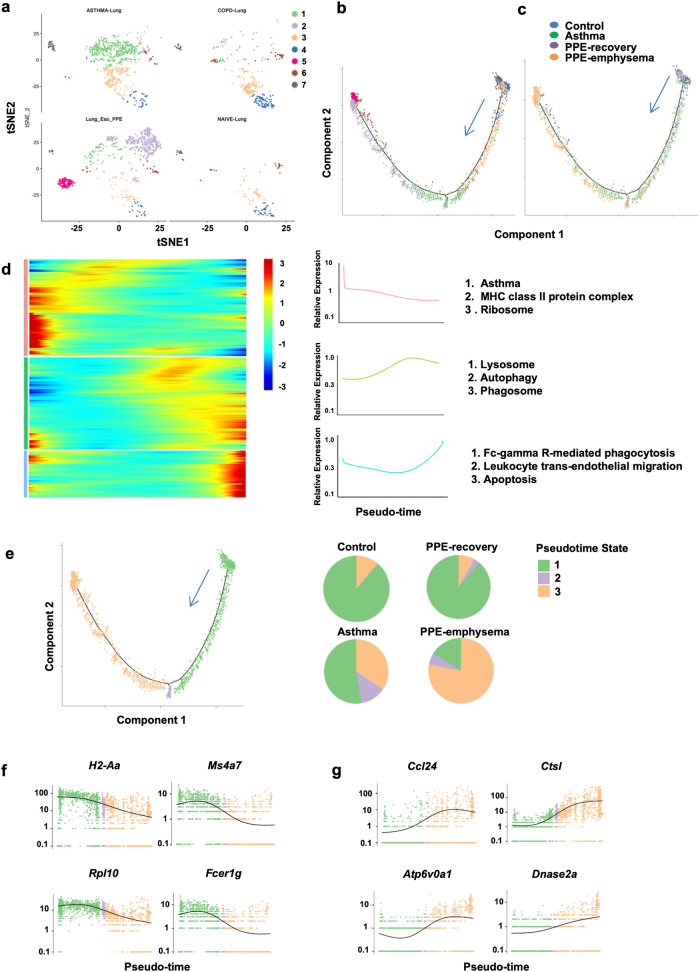
**Characterizing distinct states of eosinophils. a** Stochastic neighbor embedding (SNE) plot of eosinophil single cell subgroups. **b** The developmental pseudotime of eosinophils was determined using Monocle 2 analysis. Monocle pseudotime trajectory based on clusters. **c** Monocle pseudotime trajectory colored using different experiential models. **d** Pseudotemporal dynamics of pseudotime-associated genes within lung eosinophils. Every row is standardized based on its maximum value during the pseudotime. Aggregated expression patterns of gene sets derived from three modules across pseudotime. Enriched GO terms for gene sets from three modules are illustrated on the side. **e** Cell state and eosinophil proportions from different experimental models. **f**, **g** Significantly inhibited or activated genes during the differentiation process colored according to cell states

Single-cell transcriptome analysis of lung cells from different experimental models indicated that immune and stromal cell numbers and compositions were variable across disease models. Eosinophils accumulated in asthma and PPE-induced emphysema models, but not in the control and recovery groups (Supplementary Fig. 3a–c). Re-clustering of eosinophils resulted in the identification of seven subtypes (Fig. 3a and Supplementary Fig. 3d). Monocle was utilized to differentiate eosinophil populations along possible granulopoiesis trajectories in pseudo-time to identify the gene dynamic transformation relationship between eosinophils (Fig. 3b and Supplementary Fig. 3e).^25^ Eosinophil differentiation and maturation occurred over the course of a tightly organized trajectory, beginning from C7 and C4 cells in the control models and ending with C2 and C5 cells in emphysema models (Fig. 3c). Early stage pseudotime samples were enriched in the asthma group and was altered in the class II major histocompatibility complex, asthma, and ribosomes. Later-stage pseudotime samples were enriched in the emphysema model and had changes in the lysosome, phagosome, and autophagy processes. Interestingly, the late-stage pseudotime samples in the emphysema models were enriched for Fc gamma R-mediated phagocytosis and apoptosis signaling (Fig. 3d). These results were employed to differentiate eosinophils into three states (Fig. 3e). State 1 was defined as asthma-related eosinophils predominantly found in the asthma model, including the following genes: *H2Aa, Ms4a2*, *Fcer1g*, and *Rpl10* (Fig. 3f). State 3 eosinophils expressing *Ctsl*, *Ctsk*, *Dnase2a*, and *Atp6v0a1* emerged in the emphysema model and were linked to the lysosome, the phagosome, and autophagy processes (Fig. 3g). In addition, pseudotime analysis determined that cluster 9 was mainly enriched in states 3 and 2 in late-stage pseudotime samples, while cluster 10 was enriched in state 1 in early stage pseudotime samples. Cluster 15 was enriched in both states 1 and 3 (Supplementary Fig. 3f). These data suggested that the disease microenvironment may play a significant role in eosinophil differentiation and maturation.

### Transcription factor (TF) signatures associated with eosinophil maturation and differentiation

**Fig. 4:**
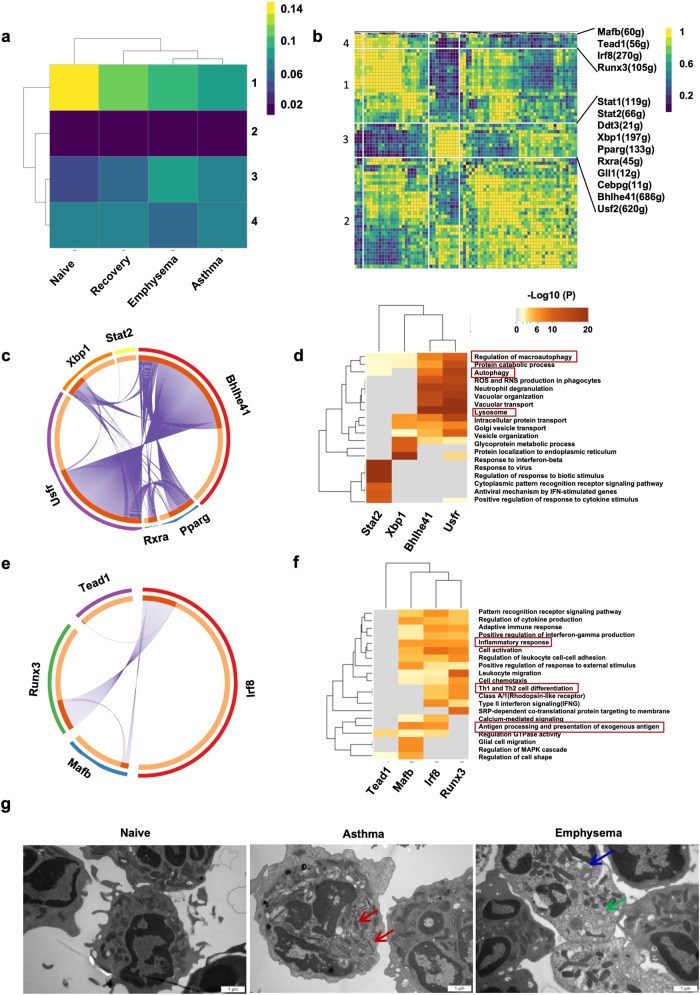
**The transcription factors associated with eosinophil maturation and differentiation. a** The heatmap portrays the regulatory activity of each module. The color gradient from blue to yellow signifies varying levels of activity, ranging from low to high, respectively. **b** The matrix of connection specificity index (CSI) underscores the correlations between regulons across diverse cell types in various experimental models. Hierarchical clustering of regulons has identified four distinct regulon modules. **c** Putative communications across differentially expressed receptors of target genes for transcription factors (TFs) within module 3. **d** Enriched GO functions of upregulated genes for module 3. **e** Putative communications between differentially expressed receptors of target genes for TFs within module 4. **f** Enriched GO functions of upregulated genes for module 4. **g** Transmission electron microscopy images of eosinophils. Red arrows indicate compact rod-like granules with electron-lucent crystalline cores. Blue arrows highlight representative eosinophil granules with reversal of core staining characterized by progressive emptying of secretory granules in the absence of granule fusions and specific release of granule contents. Green arrows indicate cytoplasmic vesicles in eosinophils derived from emphysema models

Utilizing SCENIC analysis, we evaluated precise global gene regulatory networks to delve deeper into the inherent molecular mechanisms linked to eosinophil differentiation.^26^ This exploration involved a methodical characterization of combined patterns, and the evaluation of core regulon activity scores for each regulon was conducted based on the assessment of connection specificity indices. Four primary modules were identified across the four groups (Fig. 4a, b). Module 1 contained the highest regulatory activity in control and recovery groups, along with TFs (*Fosl1*, *Jun*, *Junb*, *Rel*, and *Ets2*) that have well-documented roles in cell proliferation, differentiation, and transformation.^27^ The TFs related to NF-κB activation and angiogenesis (including *Myb*, *E2f1*, *Ets1*, *Cebpb*, *Rela*, *Nfkb1*, and *Nfkb2*) displayed elevated regulatory activity in module 2 among all experimental models.^28^ TFs, such as *Xbp1*, *Pparg*, *Bhlhe41*, and *Usf2*, were upregulated and had the highest regulatory activity in module 3 of the PPE-induced emphysema model (Fig. 4c, d and Supplementary Fig. 4).^29^ This indicates an association to eosinophil maturation and lysosome production. In contrast, the downregulated TFs (*Mafb*, *Tead1*, *Irf8*, and *Runx3*) had the lowest regulatory activity in module 4 of the PPE-induced emphysema model. This indicates that eosinophils have a reduced ability to form major histocompatibility complex class II protein complex assemblies and perform antigen presentation throughout maturation in emphysema models (Fig. 4e, f, and Supplementary Fig. 4).^30^

Indicators of eosinophil activation encompass the manifestation of membrane markers, the creation of cytoplasmic vesicles, and modifications in eosinophil granules with a decrease in electron density within the granule cores.^31^ Electron microscopy analysis of eosinophils obtained through FACS indicated that mature eosinophils in the asthma model were recognizable by their partially condensed polymorphonuclears and typical, dense, and core-containing specific granules compared to control mice. Within the emphysema models, eosinophils exhibited partial disruption of their cytoplasmic membrane, granules displaying a reversal of core staining, and a heightened occurrence of cytoplasmic vesiculation (Fig. 4g). Structures containing specific granules are associated with eosinophil maturation and activation.^32–34^ The results demonstrate that specific TFs govern eosinophil maturation and differentiation from asthma to emphysema.

### Eosinophils are required for emphysema development

**Fig. 5:**
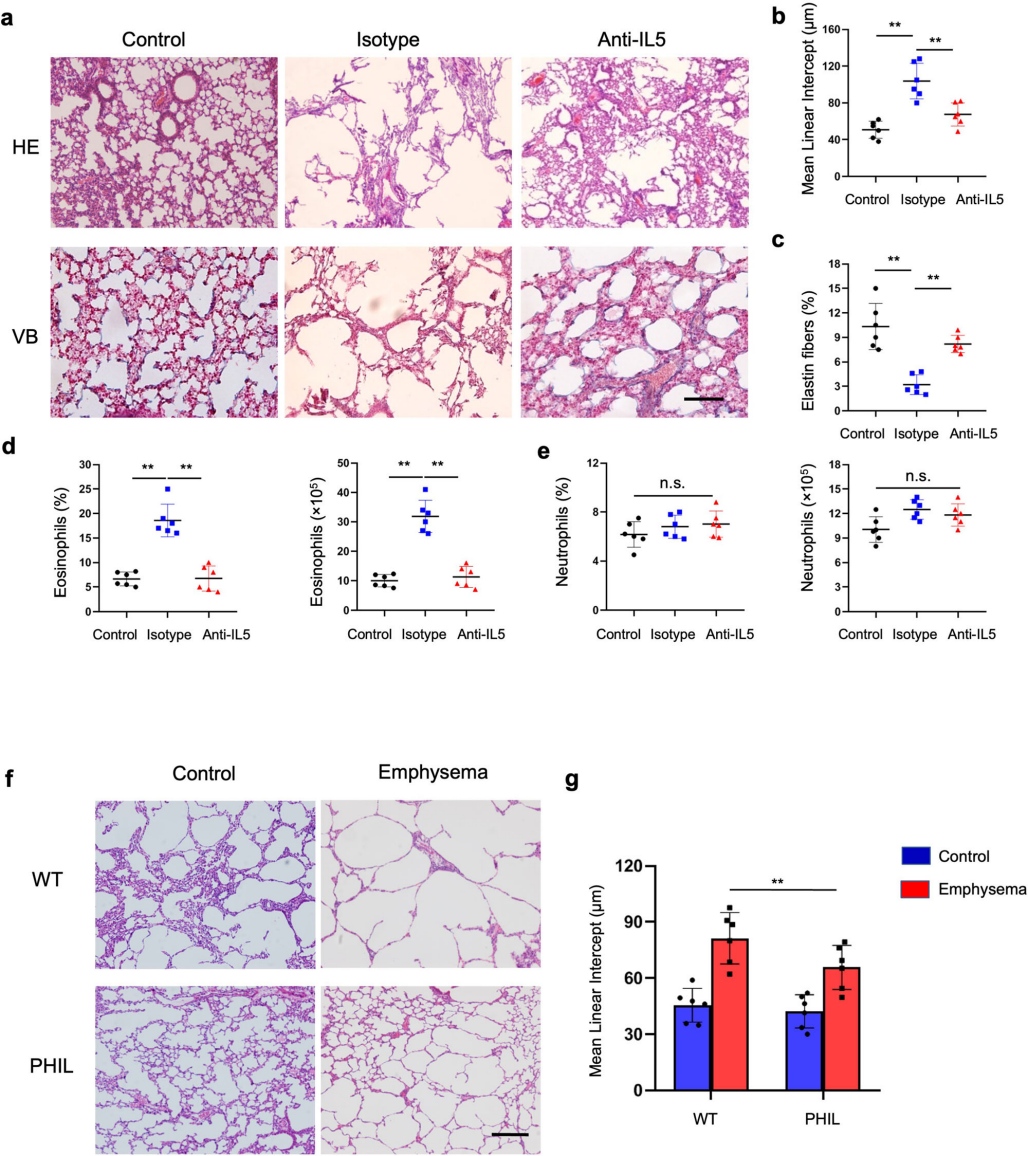
**Eosinophil deletion reduces emphysema levels in the lung parenchyma**. Mice were subjected to intratracheal instillations of porcine pancreatic elastase (PPE) on a weekly basis for 4 weeks. Each mouse received an intravenous injection of 200 μg of anti-IL-5 or control IgG 12 h before PPE treatment. **a** Pulmonary pathological sections were stained using hematoxylin and eosin (H&E) as well as Victoria Blue (VB), and subsequently visualized at the indicated magnifications (Scale bar: 100 μm). **b** The mean linear intercept and **c** elastin fiber content were quantified in the lungs of treated mice following IL-5 blockade. **d** The proportion and absolute eosinophil numbers in mouse lungs receiving the indicated treatments determined using flow cytometry. **e** Neutrophil counts in mouse lungs pretreated with anti-IL5 monoclonal antibodies or nonspecific control IgG. **f**, **g** Animals received intratracheal instillations of PPE weekly for 4 weeks. Terminal analysis was performed 3 days following the last challenge. The H&E-stained lung sections were assessed for mean intercept length (Lm), and had an increase in alveolar spaces in the lungs of wide-type (WT) mice and eosinophil-deficient (PHIL) mice. Scale bar: 200 μm. Data represent three independent experiments. Data are presented as mean ± s.e.m. ^*^P < 0.05, ^**^P < 0.01 and ^***^P < 0.001 values were determined using a two-tailed unpaired *t*-test. **b**–**g** *n* = 6 mice per group

Eosinophils were depleted through IL-5 blockade to uncover whether they were indispensable for PPE-induced emphysema (Supplementary Fig. 5a, b). Qualitative histological analysis of lung tissues indicated a significant decrease in the mean linear intercept after blocking of IL-5 (Fig. 5a, b). Elastic fibers were elevated in the lung alveolar walls after blocking IL-5 compared to the emphysema group receiving control antibodies (Fig. 5c). Moreover, endogenous IL-5 neutralization significantly lowered lung eosinophil numbers compared to those receiving control antibodies, while it had a limited impact on alveolar macrophages or neutrophils (Fig. 5d, e, and Supplementary Fig. 5c). A transgenic eosinophil-deficient mouse model (PHIL) was utilized to reduce eosinophils during emphysema development specifically. These data were complemented by a genetic depletion model resulting in eosinophil cell death, with PHIL mice endogenously expressing the diphtheria toxin receptor under the control of the eosinophil peroxidase promoter.^35^ Eosinophil reduction resulted in a notable decrease in emphysema in PHIL mice compared to wild-type controls (Fig. 5f, g). These data outline that eosinophils play a crucial role in emphysema development.

### CTSL levels are increased in the eosinophils of PPE-induced emphysema

**Fig. 6:**
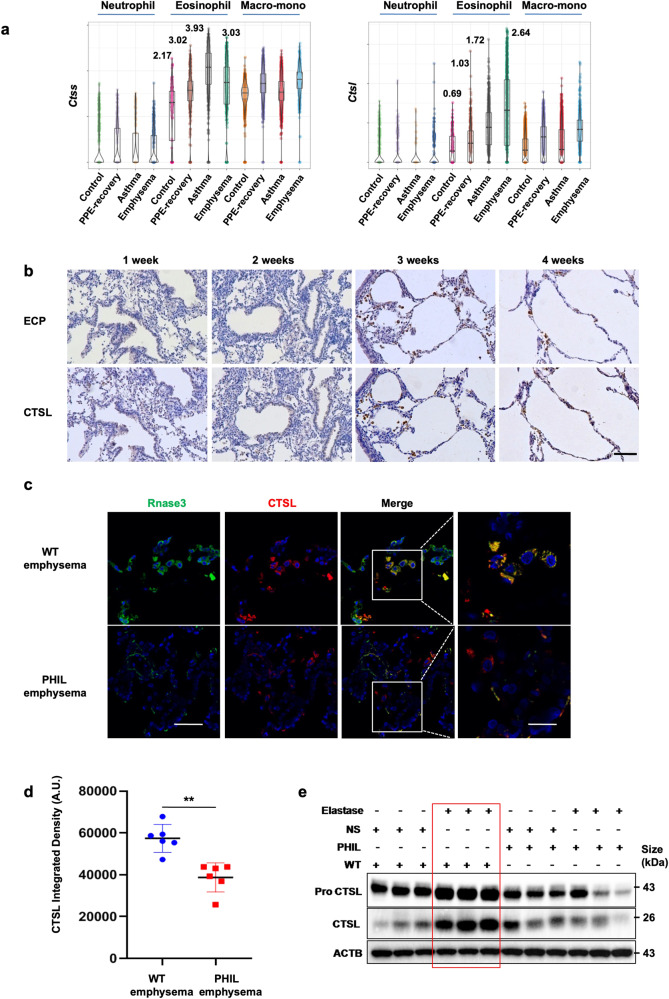
**Cathepsin L levels increased in the eosinophils of PPE-induced emphysema. a** Expression of two typical eosinophil granule genes. **b** Animals were administered intratracheal instillations of PPE once a week for 4 weeks. The mice were euthanized on day 3 following PPE challenge, samples were collected, and immunohistochemistry experiments on sections for ECP and CTSL were performed. Scale bars represent 100 μm. **c** Animals received intratracheal instillations of PPE weekly for 4 weeks and were sacrificed on day three following the last challenge. Immunofluorescence analysis was conducted on lung tissue samples obtained from both WT and PHIL mice to assess CTSL, Rnase3 (ECP), and DAPI labeling. Illustrated here are side-by-side images of the two groups of mice. The original magnifications are as follows: the 1st to 3rd lanes depict standard views, and the 4th shows an enlarged image of the indicated region (Scale bars for low and high magnifications are 50 μm and 20 μm, respectively). **d** CTSL fluorescence intensity was assessed, and arbitrary units were indicated. **e** Immunoblotting was performed for Pro-CTSL and CTSL, with ACTB included as a control for lungs. The data represent three independent experiments and are expressed as the mean ± s.e.m. *P < 0.05, **P < 0.01 and ***P < 0.001 determined using a two-tailed *t-*test. **b**–**e**, *n* = 6 mice per group

Several studies have demonstrated that proteases are essential in extracellular matrix degradation and remodeling.^36^ We detected *Ctsl*, *Ctss*, *Mmp9*, and *Elane* expression in different cell groups across emphysematous models. *Ctsl* (but not *Ctss*, *Elane*, or *Mmp9*) was highly expressed in eosinophils from emphysema models (Fig. 6a and Supplementary Fig. 6a). Eosinophils accumulated in the lung alveoli after the third PPE challenge (Supplementary Fig. 6b). The transition in CTSL dynamics was akin to that of eosinophils during emphysema development (Fig. 6b). Immunofluorescence analysis demonstrated that CTSL was expressed in eosinophils and increased in emphysematous lung tissues (Fig. 6c, d). Western blot analysis showed that CTSL protein levels significantly increased in PPE-induced emphysematous lungs of wild-type mice but not in PHIL mice (Fig. 6e). These results imply that eosinophils are the primary source of pulmonary CTSL throughout emphysema development.

### CTSL derived from eosinophils is critical for PPE-induced emphysema

**Fig. 7:**
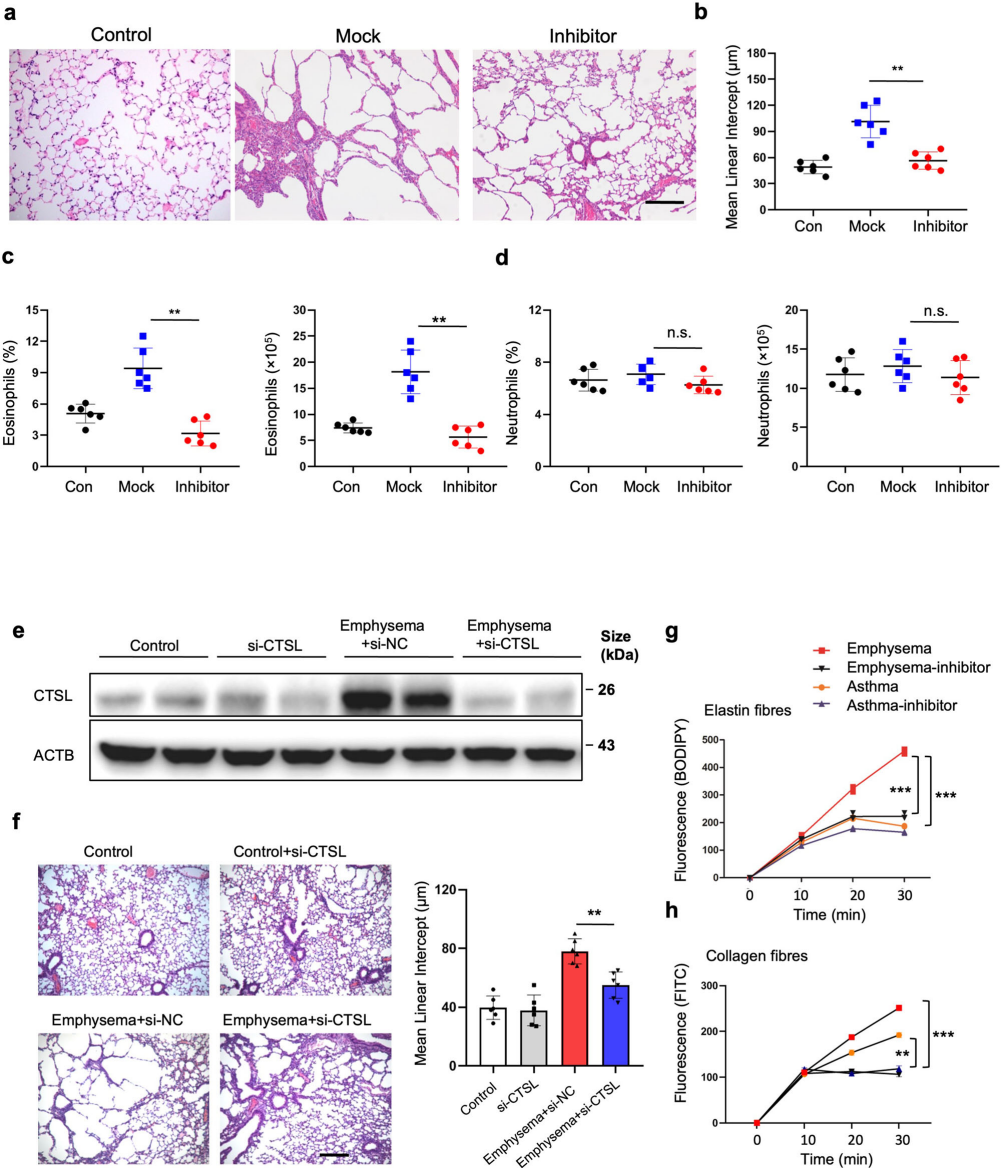
**Cathepsin L derived from eosinophils are crucial for emphysema**. **a**, **b** Mean linear intercepts were examined in mice lungs pretreated with 500 μg CTSL inhibitor (SID 26681509, quarterhydrate) or vehicle control 4 times 12 h before the first instillation of PPE. Scale bar: 200 μm. **c**, **d** Eosinophil counts and neutrophil counts were examined in mice lungs. **e** Mice were administered AAV-CTSL (5 × 10^12^ vg/mL) in 60 μL PBS via intratracheal (i.t.) administration to cause CTSL expression in lungs 14 days before emphysema model establishment. Control mice were treated with controladeno-associated virus. The animals received intratracheal instillations of PPE or PBS weekly for 3 weeks on day 3 after the third challenge. After being sacrificed, CTSL expression in lung tissues of the control, control-si-CTSL, emphysema-si-NC, and emphysema-si-CTSL groups were examined. **f** Mean linear intercept in the treated mice lungs. Scale bar: 200 μm. **g** Cathepsin enzymatic activity was evaluated at 5-min intervals, specifically to monitor the generation of BODIPY FL-labeled fluorescent elastin fragments from self-quenching BODIPY FL-conjugated bovine neck ligament elastin. **h** Cathepsin activity was also assessed for the generation of fluorescein isothiocyanate (FITC) from FITC-labeled type I collagen. These data were obtained from three independent experiments and are highlighted as the mean ± s.e.m. *P < 0.05, **P < 0.01 and ***P < 0.001 were analyzed using a two-tailed *t-*test. Panels **a**–**f**, *n* = 6 mice per group

Many ECM proteins also operate as CTSL substrates.^37^ A CTSL inhibitor (quarterhydrate, SID 26681509) was utilized further to uncover the functions of CTSL in emphysema development.^38^ Mice administered the CTSL inhibitor showed significantly decreased emphysema severity compared to the control group (Fig. 7a, b and Supplementary Fig. 7a). The number of eosinophils (but not alveolar macrophages or neutrophils) significantly dropped after CTSL blockade (Fig. 7c, d and Supplementary Fig. 7b). *Ctsl* and *Mmp12* expression was reduced after inhibitor treatment. In contrast, *Ctss*, *Mmp9*, and *Elane* expression did not significantly differ from that in the mock group (Supplementary Fig. 7c). Additionally, adenoviral gene delivery using AAV-CTSL substantially downregulated CTSL levels (Supplementary Fig. 7d, e and Fig. 7e) and emphysema severity in the lungs compared with the control group (Fig. 7f). Furthermore, the levels and percentage of eosinophils (but not alveolar macrophages or neutrophils) in the lungs significantly decreased after AAV-CTSL administration (Supplementary Fig. 7f). These results support the crucial role of CTSL in PPE-induced emphysema.

Isolated eosinophils obtained from the emphysema models were subjected to testing using a BODIPY-tagged elastin substrate. This substrate exhibited fluorescence upon the cleavage of the elastin molecule, indicating its active and accessible state. In contrast, eosinophils derived from asthma models had reduced elastolytic capacity. Additionally, the elastolytic ability of eosinophils isolated from the emphysema models was limited by the CTSL inhibitor (Fig. 7g). Proteases released from eosinophils in the emphysema models exhibited robust collagenase activity, while those released from the asthma models lacked this activity (Fig. 7h). These data indicate that CTSL derived from eosinophils promotes ECM destruction during emphysema development.

### CTSL expression in eosinophils is linked to emphysema in COPD patients

**Fig. 8:**
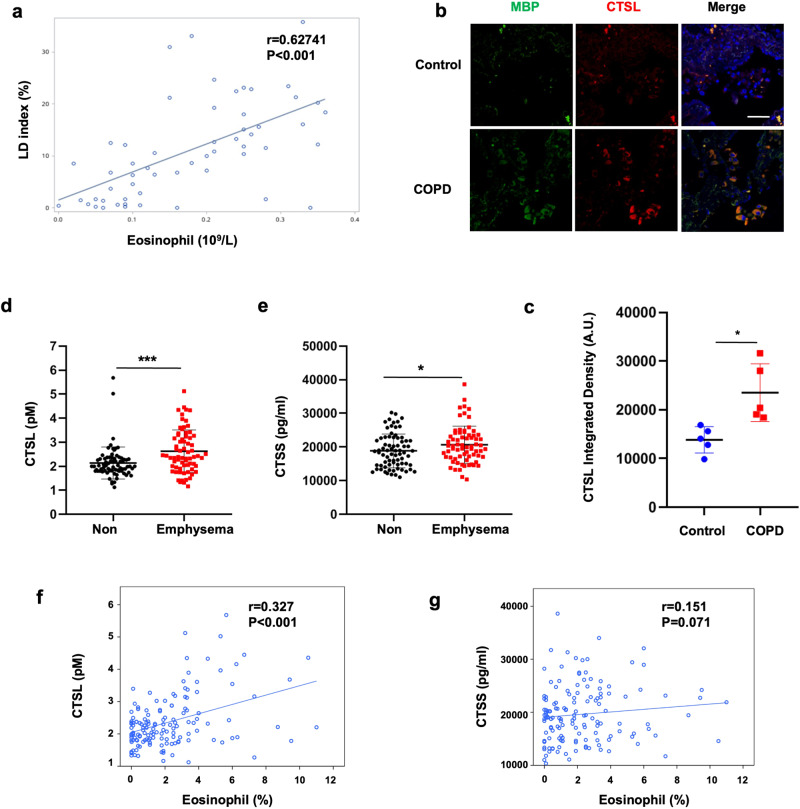
**CTSL expression in eosinophils is linked to emphysema in patients with COPD. a** The association between lung density index and blood eosinophil levels was analyzed through Spearman’s correlation (*n* = 56, *r* = 0.627, *P* < 0.0001). **b** Lung tissue from healthy controls and COPD patients stained for CTSL (red), MBP (green), and DAPI (blue), with CTSL staining intensity quantified. **c** CTSL fluorescence intensity, arbitrary units are shown. Scale bar: 50 μm. **d**, **e** Comparison of serum levels of CTSL (*P* < 0.001) and CTSS (*P* = 0.0046) across patients with COPD with or without emphysema. **f**, **g** Spearman’s rank correlation analysis of the relationship between total serum CTSL (*r* = 0.327, *P* < 0.0001), CTSS (*r* = 0.151, *P* = 0.071), and eosinophil levels. Data represent the mean ± s.e.m. *P < 0.05, **P < 0.01, ***P < 0.001 values were determined using the Mann–Whitney *U*-test. **b**, *n* = 5 for each group; **c**, **d**, *n* = 74 and 70 for the non-emphysema and emphysema groups, respectively. **e**, **f**, *n* = 144 for total COPD patients

Clinical data were obtained from 56 COPD patients to determine the association between eosinophils and emphysema (Supplementary Table 1). The lung emphysema index determined from chest computed tomography scan data was positively correlated to blood eosinophil levels (*r* = 0.627, *P* < 0.0001) (Fig. 8a). Elevated eosinophil levels were positively associated with emphysema (defined as LAA% ≥ 10%) in the multivariable logistic regression with covariates adjusted (OR = 46.82, 95% CI = 6.12-358.33, *P* < 0.001) (Supplementary Table 2). These data indicated that emphysema in patients with COPD is linked to high blood eosinophil count. Immunofluorescence analysis demonstrated that CTSL was primarily localized to eosinophils, and CTSL was elevated in alveolar tissue from COPD patients compared to healthy controls (Fig. 8b, c). Moreover, patients with emphysema had increased serum CTSL and CTSS levels compared to patients lacking emphysema (*P* < 0.001 for CTSL, *P* = 0.0046 for CTSS) (Fig. 8d, e). As anticipated, blood eosinophil levels were positively related to serum CTSL levels in COPD patients but not CTSS patients (*P* < 0.0001 for CTSL, *P* = 0.071 for CTSS) (Fig. 7f, g). In contrast, neutrophils did not correlate with either CTSL or CTSS levels (Supplementary Fig. 8a, b). RNA-seq analysis demonstrated that CTSL was highly expressed in eosinophils derived from patients with emphysema and asthma-COPD overlap (Supplementary Fig. 8c, d). No significant difference was found in the CTSL levels between healthy controls and patients with eosinophilic pneumonia (Supplementary Fig. 8e). These findings indicate that increased CTSL levels in COPD patients may influence the development of emphysema.

## Discussion

This study examined the role and mechanism of action of eosinophils in emphysema pathophysiology. A specific eosinophil subtype was identified, with enriched proteins found in the endoplasmic reticulum, lysosomes, and phagosomes, which might be essential for the development of emphysema. CTSL was predominantly enhanced in eosinophils but not in neutrophils in emphysema models. Mechanistic studies utilizing CTSL inhibitors and an eosinophil-deficient mouse model verified the role of CTSL in ECM degradation and emphysema development. Our results facilitate new insights into the role of eosinophils in promoting emphysema in COPD, possibly through the secretion of CTSL proteinase. To date, there have been no approved targeted therapies for emphysema in COPD patients. Our study provides a potential target for emphysema treatment.

Patients with asthma possessing elevated blood and airway eosinophil levels typically do not develop emphysema, which is a puzzling progression. Airspace enlargement in patients with asthma is characteristic of air trapping caused by small airway disease rather than alveolar wall destruction.^39^ Asthma is a significant risk factor for COPD, and patients with asthma may need more time to develop emphysema. However, previous studies have been limited by the lack of long-term follow-up.^40^ Alternatively, the differences in eosinophil phenotype and function in COPD and asthma may allow for an explanation.^41^ Systematic ultrastructural studies identified four types of eosinophils based on their activation state: the inactive state characterized by intact cytoplasmic granules, piecemeal degranulation with changes in the specific intracytoplasmic granules, cytolysis with ruptured membrane, and partially dissolved cytoplasm and apoptosis.^34,41^ ScRNA-seq may assist in identifying new cell subgroups to reveal unclarified cell functions. Our results indicate that compared to eosinophils in asthma models, emphysema-related eosinophils are closely linked to the maturation and terminal differentiation of eosinophils from immature progenitors throughout emphysema development.

The potential functions of eosinophils in COPD have remained unclear, and clinical trials have given inconsistent results.^15–17^ Clinical trials of IL-4R mAb (dupilumab, NCT04456673) and thymic stromal lymphopoietin mAb (tezepelumab, NCT05507242) are still ongoing. Most clinical trials utilize acute exacerbation as the primary endpoint as opposed to lung function or emphysema. Thus, to engineer strategies targeting eosinophils in the initiation and development of COPD or emphysema, long-term studies using lung function as the primary outcome may be needed. Notably, for mechanistic studies, a transgenic mouse model of chronic type 2 pulmonary inflammation (I5/hE2) has uncovered an eosinophil-dependent mechanism resulting in airspace enlargement.^20^ In our current study, we illuminated the critical role of eosinophil-derived CTSL in emphysema development.

Eosinophils in emphysematous lungs are linked to lysosomes, phagosomes, and autophagy, suggesting proteinase production and ECM degradation by eosinophils. Eosinophils are the main cause of pulmonary CTSL in emphysema models. CTSL plays a crucial role in diverse cellular processes, encompassing protein degradation, autophagy, cell death, ECM remodeling, and immune responses.^37^ Its involvement spans the onset and progression of various human diseases, including cancer, cardiovascular diseases, and inflammatory disorders, alongside its physiological functions in normal cells.^36,42,43^ Recent research has unveiled CTSL’s contribution to membrane fusion and the release of virus genomes during SARS-CoV-2 infection through the proteolytic activation of the spike protein.^44^ Elevated CTSL levels in the bloodstream are linked to compromised lung function and increased bronchoalveolar fluid, while patients with emphysema display heightened CTSL levels.^45,46^ Our investigation showcases that eosinophils actively partake in COPD pathogenesis and emphysema development by producing CTSL.

In conclusion, these findings outline the first reference map for differentiating mature eosinophil transcriptional states across diverse disease microenvironments. Eosinophil-derived CTSL is crucial for ECM degradation and remodeling, resulting in emphysema development. This study underscores the potential of CTSL as a therapeutic target in emphysema patients.

## Materials and methods

### Patients and lung samples

A total of 144 patients with physician-diagnosed COPD were recruited for this study. All participants underwent a post-bronchodilator lung function examination to confirm their diagnosis, and 56 patients with COPD volunteered for chest computed tomography (Supplementary Table 1). The emphysema index (LAA%) was derived from chest computed tomography scan data for quantitative assessment of the severity of emphysema (**Supplementary Methods**). We additionally gathered data from five individuals who had passed away and donated their lungs, along with five COPD patients who underwent lung transplantation. The human studies were granted approval by the Ethical Committee on Clinical Research of the China-Japan Friendship Hospital (2019-106-K74). All participants provided written informed consent for their involvement.

### Animals and experimental models

PHIL mice were provided by Professor James Lee at the Mayo Clinic (AZ).^47^ Pulmonary eosinophils were removed as previously described.^19^ Briefly, PHIL mice expressing the diphtheria toxin receptor under the control of the eosinophil peroxidase promoter were subjected to eosinophil cell death upon expression of eosinophil peroxidase in the eosinophil lineage-committed cells. C57BL/6 mice were obtained from Charles River Animal Co. Ltd. All mice were maintained under specific pathogen-free conditions at the Laboratory Animal Center of Zhejiang University and Capital Medical University. The emphysema mouse model was developed using intratracheal instillations of 0.5 or 2 U of PPE (Sigma, St. Louis, MO, USA) dissolved in 100 µL PBS as described previously.^6^ The ovalbumin (Sigma)-induced allergic airway inflammation model was established based on a previous method.^48^ All experimental protocols were approved by the Ethical Committee for Animal Studies of Capital Medical University (AEEI-2021-272) and Zhejiang University (ZJU20210005).

### ScRNA-seq

We prepared single-cell suspensions and loaded them onto the Chromium Single Cell Controller instrument (10×Genomics, Pleasanton, CA, USA) to create single-cell gel beads in emulsions for subsequent sequencing and analysis. Comprehensive procedures, including relevant accession codes and references, can be found in the online version of this paper.

### Histopathology and immunofluorescence staining

Mouse lungs were fixed at a pressure of 20 cmH_2_O, and 5 µm-thick tissue sections were stained with H&E to assess the mean linear intercept. Masson’s trichrome and elastin staining were conducted to evaluate the elastic and collagen fibers through image analysis (Image-Pro Plus 6.0). Immunofluorescent staining proceeded by incubating sections with primary antibodies at 4 °C overnight and staining with corresponding fluorochrome-conjugated secondary antibodies (Supplementary Table 3). Fluorescence was detected using an Olympus FV3000 confocal laser-scanning microscope.

### Serum CTSL and CTSS measurements

Immunoblotting was conducted for CTSL, pro-CTSL, and β-actin (Supplementary Table 3). CTSL activity was assessed using a Cathepsin L Activity Assay Kit (Abcam, ab65306) and quantified using the CL Substrate Ac-FR-AFC kit (Abcam, ab157769). Serum CTSS concentrations were examined using a CTSS ELISA kit (Invitrogen, EHCTSS).

### Fluorescence elastin degradation and collagen degradation assays

The DQ elastin substrate, included in the EnzChek Elastase Assay kit (E-12056) from Thermo Fisher Scientific and the Collagenase Assay Kit (Fluorometric, K490) from BioVision, were used to assess CTSL’s elastolytic capacity. Eosinophils were prepared at a final concentration of 5 × 10^5^ in a reaction buffer volume of 200 µL/well within a black 96-well plate. CTSL activity was measured by monitoring fluorescence emission at 515 nm (BODIPY) or 520 nm (fluorescein isothiocyanate) using a Spectra Max Gemini fluorescent 96-well plate reader at 5-min intervals. Inhibition experiments were carried out as previously described, with the addition of quarterhydrate (MCE, SID 26681509) before substrate introduction. Data analysis was conducted utilizing the SoftMax Pro software.

### Transmission electron microscopy

Isolated cells were rapidly fixed and dehydrated utilizing an acetone gradient series. Samples were infiltrated, embedded in resin, and sectioned into 70 nm segments. Sample sections were stained and examined using a transmission electron microscope (JEOL JEM-1400; Tokyo, Japan).

### Flow cytometry

Mouse lung tissues were processed to yield single-cell suspensions, following previously documented methods.^21^ BD Biosciences provided anti-mouse antibodies targeting CD11b, CD11c, Ly6G, and Siglec-F. The entire lung tissue underwent RBC lysis buffer treatment, followed by PBS washing and staining with respective antibodies (Supplementary Table 3). Flow cytometric analysis was performed using a BD LSRFortessa flow cytometer (BD Immunocytometry Systems), and the resulting data were subjected to analysis using FlowJo software (Tree Star).

### IL-5 or CTSL blockade

In mice, the inhibition of IL-5 was accomplished by administering an intravenous injection of an anti-IL-5 antibody (TRFK5; BD Pharmingen) or an isotype-matched control antibody (R3-34; BD Pharmingen) at a dose of 200 g/mouse, 24 h before PPE challenge. For CTSL inhibition, mice were pre-treated with 500 μg of CTSL inhibitor (SID 26681509, quarterhydrate) or a vehicle control, 12 h before PPE instillation. On the 3rd day after the final PPE challenge, the mice were euthanized, and samples were collected for subsequent analysis.

### Adenovirus gene delivery

Recombinant adeno-associated viruses containing the mouse *CTSL* gene were procured from Hanheng Biotechnology Co., Ltd. (Shanghai, China). Mice were anesthetized using tribromoethanol 14 days prior to the establishment of the emphysema model and intratracheal administration of AAV2/5-m-Ctsl-EGFP (1.5 × 10^12^ vg/mL) in 60 μL PBS to reduce CTSL expression in the lungs. The adeno-associated virus AAV2/5-EGFP NC (1.5 × 10^12^ vg/mL) was employed as a negative control (Supplementary Table 4). The effectiveness of the fusion protein was assessed through Western blotting.

### RNA fluorescence in situ hybridization (FISH)

We conducted a FISH assay to identify and pinpoint the presence of *Rnase2a*, *C1qa*, and *Alox15* mRNA within clusters 9, 10, and 15. Fluorescently labeled probes were custom-synthesized by ServiceBio Company (Wuhan, China). RNA FISH assays were executed using the Servicebio™ FISH kit (GF010-50T, Servicebio Company) in line with the manufacturer’s guidelines. Subsequently, ECP was immunodetected. In situ hybridization was followed by overnight incubation with primary anti-ECP antibodies (orb156688, Biorbyt) at 4 °C and detection utilizing Donkey anti-rabbit-Alex Fluor^TM^ 555 (A-31572, Invitrogen). Images were captured utilizing an Olympus FV3000 confocal laser-scanning microscope with a 60x objective.

### Statistical analysis

Categorical variables were represented as frequencies and proportions within clinical data. For continuous variables, their adherence to normal distribution was assessed, and they are presented as means ± standard deviation or as medians (interquartile range) as appropriate. To compare differences between groups, *t*-tests or Mann–Whitney U-tests were employed for normally distributed or non-normally distributed continuous variables, respectively. Categorical variables were analyzed using Pearson’s chi-squared test or Fisher’s exact test to determine group disparities. Spearman’s correlation analysis was utilized to explore the connection between lung density index and blood eosinophil count. Multivariate logistic regression was applied to examine the relationship between eosinophil count and emphysema. Experimental animal data values were expressed as means ± SEM, with each experiment independently replicated at least three times. GraphPad Prism software was employed for statistical analysis, utilizing two-tailed *t*-tests to determine statistical significance. Statistical significance was established at *P* < 0.05.

### Supplementary information

Supplementary materials

## Data Availability

The single-cell RNA and Bulk RNA sequencing data from mice generated in this study have been deposited in the GEO database under accession code “GSE 237749”; Bulk RNA sequencing data from humans is available under accession number “HRA005107” in the GSA (Genome Sequence Archive) database. Matrices or other relevant data are available from the corresponding author upon a reasonable request.
