# Correction: Eosinophils promote pulmonary matrix destruction and emphysema via Cathepsin L

**DOI:** 10.1038/s41392-023-01698-9

**Published:** 2023-11-27

**Authors:** Xia Xu, Tao Yu, Lingling Dong, Rainer Glauben, Siyuan Wu, Ronghua Huang, Shiwei Qumu, Chenli Chang, Jing Guo, Lin Pan, Ting Yang, Xin Lin, Ke Huang, Zhihua Chen, Chen Wang

**Affiliations:** 1 Department of Immunology, School of Basic Medical Sciences, Capital Medical University, Beijing, China; 2 Department of Pulmonary and Critical Care Medicine, China-Japan Friendship Hospital, Beijing, China; 3 Key Laboratory of Respiratory Disease of Zhejiang Province, Department of Respiratory and Critical Care Medicine, Second Affiliated Hospital of Zhejiang University School of Medicine, Hangzhou, Zhejiang China; 4 Department of Gastroenterology, Infectious Diseases, and Rheumatology, Campus Benjamin Franklin, Charité-University Medicine Berlin, Berlin, Germany; 5 Institute of Respiratory Medicine, Chinese Academy of Medical Science, Beijing, China; 6 Institute of Clinical Medical Sciences, China-Japan Friendship Hospital, Beijing, China; 7 Institute for Immunology, Tsinghua University School of Medicine, Beijing, China

**Keywords:** Respiratory tract diseases, Inflammation

Correction to: *Signal Transduction and Targeted Therapy* 10.1038/s41392-023-01634-x, published online 11 October 2023

After online publication of the article^1^, the authors noticed that they made a mistake by arranging the figure of other group (Figure 7a, Inhibitor Group) into 5f during typesetting. In addition, We also made frame surrounding the blot of Figure 7e to meet the requirements of Author Checklist. The correct figures were provided as follows.

Figure 5
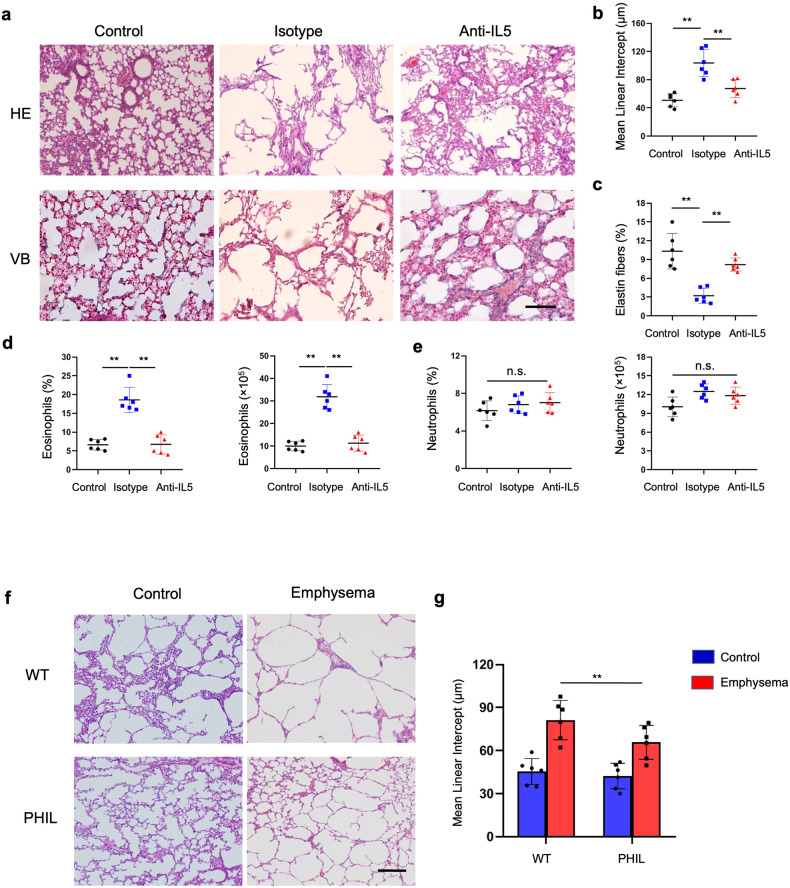


Figure 7
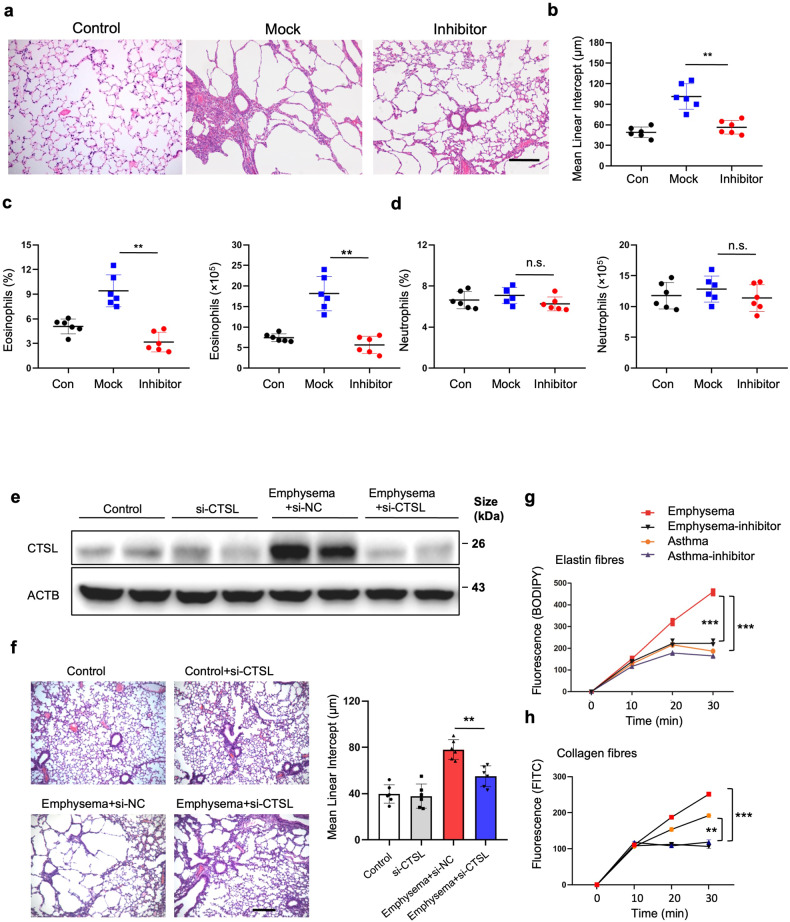


The original article has been corrected.
