# Supplementary material for: Eosinophils promote pulmonary matrix destruction and emphysema via Cathepsin L
Source: Signal Transduct Target Ther. 2023 Oct 11;8:390. doi: 10.1038/s41392-023-01634-x (PMC10564720; doi:10.1038/s41392-023-01634-x)

**Supplementary Materials for**

**Eosinophil promotes pulmonary matrix destruction and emphysema via Cathepsin L**

Xia Xu^1^, Tao Yu^2^, Lingling Dong^3^, Rainer Glauben^4^, Siyuan Wu^1^, Ronghua Huang^1^, Shiwei Qumu^2,5^, Chenli Chang^5^, Jing Guo^6^, Lin Pan^6^, Ting Yang^2,5^, Xin Lin^7^, Ke Huang^2,5^*, Zhihua Chen^3^*, Chen Wang^1,2,5^*

^1^ Department of Immunology, School of Basic Medical Sciences, Capital Medical University, Beijing, China.

^2^ Department of Pulmonary and Critical Care Medicine, China-Japan Friendship Hospital, Beijing, China

^3^ Key Laboratory of Respiratory Disease of Zhejiang Province, Department of Respiratory and Critical Care Medicine, Second Affiliated Hospital of Zhejiang University School of Medicine, Hangzhou, Zhejiang, China

^4^ Department of Gastroenterology, Infectious Diseases, and Rheumatology, Campus Benjamin Franklin, Charité-University Medicine Berlin, Berlin, Germany

^5^ Institute of Respiratory Medicine, Chinese Academy of Medical Science, Beijing, China

^6^ Institute of Clinical Medical Sciences, China-Japan Friendship Hospital, Beijing, China

^7^ Institute for Immunology, Tsinghua University School of Medicine, Beijing, China

**Author Information:**

*To whom correspondence should be addressed: Ke Huang ([huangke_zryy@163.com](mailto:huangke_zryy@163.com)) or Zhihua Chen ([zhihuachen@zju.edu.cn](mailto:zhihuachen@zju.edu.cn)) or Chen Wang ([cyh-birm@263.net](mailto:cyh-birm@263.net))

**This file includes:**

Materials and Methods

Supplementary Figures. S1 to S8

Supplementary Tables S1 to S4

Supplementary original and uncropped films of Western blots

**Supplementary Materials and Methods**

**Chest CT scan and emphysema assessment:**

Among all volunteers, 56 volunteers accepted chest CT scan in China-Japan Friendship Hospital (Beijing, China). Chest CT images were captured while volunteers laid supine at suspended full inspiration from apex to base of the lungs. All images were evaluated using The Digital Lung software (Dexin, China). Emphysema was quantified as the percentage ratio of low-attenuation volume below a threshold of -950 Hounsfield units to the total lung volume (Emphysema index, LAA%). Patients were considered to have emphysema if the LAA% was greater than 10%.

**Single-cell RNA sequencing**

Single-cell RNA-seq libraries were prepared using a Chromium Single-cell 3' Reagent kit, version 2, according to the manufacturer’s protocol. Single-cell suspensions were loaded on the Chromium Single Cell Controller Instrument (10×Genomics, Pleasanton, CA, USA) to generate single-cell gel beads in emulsions (GEMs). Then these libraries were sequenced on the Illumina sequencing Platform (novaseq 6000), and 150 bp paired-end reads were generated. The Cell Ranger software pipeline (version 3.1.0) provided by 10×Genomics was used to demultiplex cellular barcodes, map reads to the genome, align transcriptomes using the STAR aligner, and down-sample reads as required to generate normalized aggregate data across samples, producing a matrix of gene counts versus cells. We processed the unique molecular identifier(UMI) count matrix using the R package Seurat (version 3.3.1). To remove low-quality cells and likely multiple captures, we applied a criterion to filter out cells with UMI/gene numbers out of the limit of mean value +/- 2-fold of standard deviations, assuming a Gaussian distribution of each cells' UMI/gene numbers, and discarded low-quality cells where >10% of the counts belonged to mitochondrial genes. Library size normalization was performed with Normalize Data function in Seurat to obtain the normalized count. To remove the batch effects in single-cell RNA-sequencing data, the mutual nearest neighbors(MNN) was performed with the R package batchelor. Graph-based clustering was performed to cluster cells according to their gene expression profile using the FindClusters function in Seurat. Cells were visualized using a 2-dimensional t-SNE algorithm with the RunTSNE function. We used the FindAllMarkers function (test.use = bimod) in Seurat to identify marker genes of each cluster. FindAllMarkers identified positive markers for a given cluster compared with all other cells. Differentially expressed genes(DEGs) were identified using the FindMarkers function(test.use = MAST) in Seurat. P-value<0.05 and |log2foldchange| > 0.58 was set as the threshold for significantly differential expression. GO enrichment and KEGG pathway enrichment analysis of DEGs were respectively performed using R based on the hypergeometric distribution.

**Pseudotime Analysis**

The developmental pseudotime was determined with the Monocle2 package. The raw count was first converted from the Seurat object into CellDataSet object with the import CDS function in Monocle. The differential GeneTest function of the Monocle2 package was used to select ordering genes (qval< 0.01), which were likely to be informative in the ordering of cells along the pseudotime trajectory. The dimensional reduction clustering analysis was performed with the reduce Dimension function, followed by trajectory inference with the order Cells function using default parameters. Gene expression was plotted with the plot_genes_in_pseudotime function to track changes over pseudo-time.

**SCENIC Analysis**

The SCENIC analysis was run using the motifs database for RcisTarget and GRNboost (SCENIC^1^ version 1.1.2.2, corresponding to RcisTarget 1.2.1 and AUCell 1.4.1) with the default parameters. In detail, we identified TF binding motifs over-represented on a gene list with RcisTarget package. The activity of each group of regulons in each cell was scored by AUCellpackage. To evaluate the cell type specificity of each predicted regulon, we calculated the regulon specificity score (RSS) based on the Jensen-Shannon divergence (JSD), a measure of the similarity between two probability distributions. Specifically, we calculated the JSD (Jensen-Shannon divergence) between each vector of binary regulon activity overlaps with the assignment of cells to a specific cell type.^2^ The connection specificity index (CSI) for all regulons was calculated with the scFunctions (<https://github.com/FloWuenne/scFunctions/>) package.

## Quantitative PCR

Total RNA was isolated using TRIzol (Invitrogen) and then 1 μg of RNA was reverse transcribed using PrimeScript™ RT Master Mix (Takara, RR036A). qPCR was performed using Power SYBR Green PCR Master Mix (TaKaRa, RR420A). The amounts of transcripts were normalized to those of GAPDH. The primers used in this study are listed in Supplementary Table 4

**Reference**

1 Aibar, S. et al. SCENIC: single-cell regulatory network inference and clustering. *Nat Methods* ***14***, 1083-1086 (2017).

2 Suo, S. et al. Revealing the Critical Regulators of Cell Identity in the Mouse Cell Atlas. *Cell Rep* ***25***,1436-1445 (2018).

**Supplementary Figures:**

**
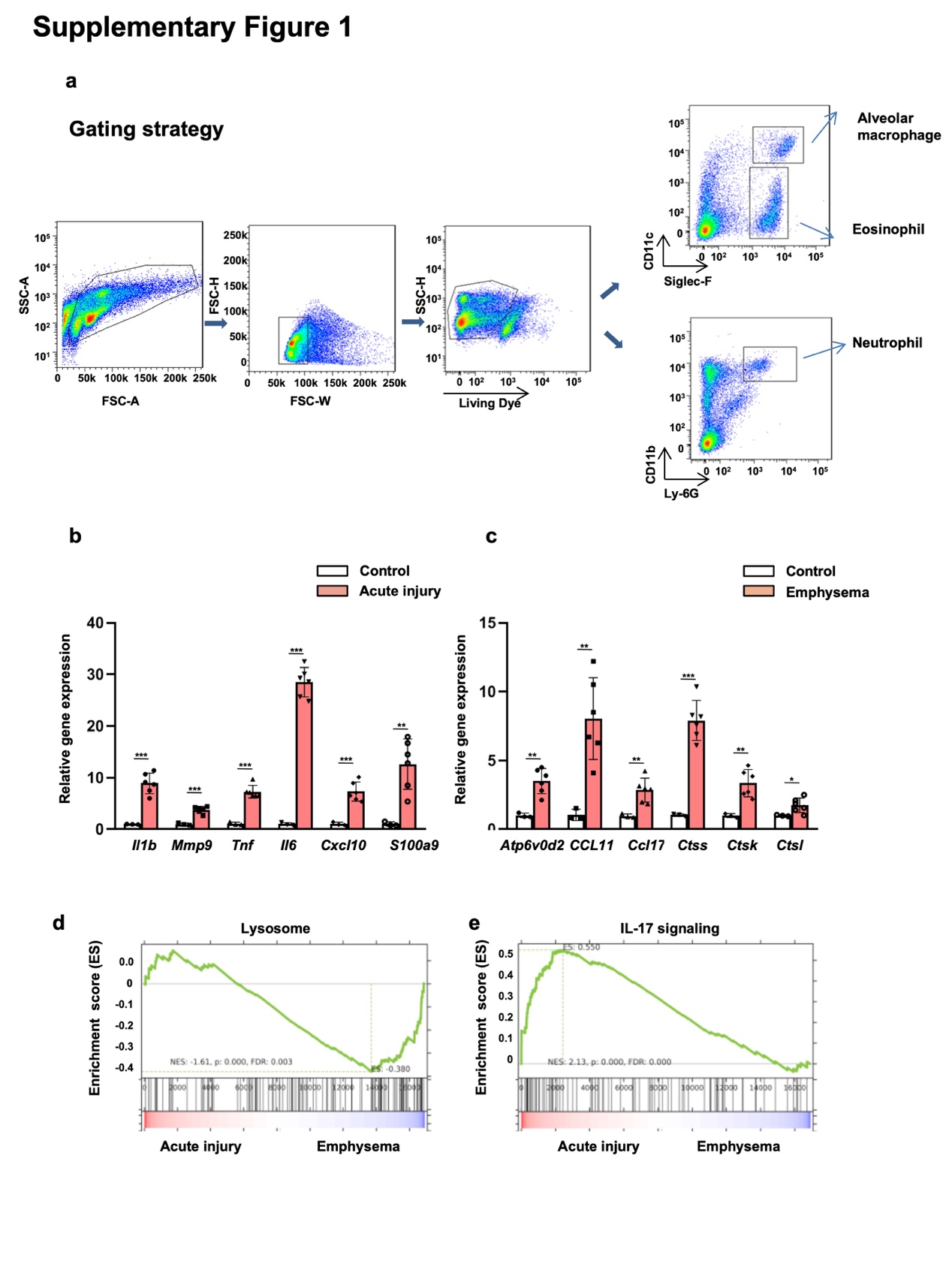
**

**Figure S1. The switch from acute neutrophilic inflammation to chronic eosinophilic inflammation in PPE-induced emphysema models.**

(a) Gating strategy to analyze by flow cytometry for alveolar macrophage (CD11c^+^SiglecF^+^), eosinophil (CD11c^-^SiglecF^+^) and neutrophil (CD11b^+^SiglecF^+^) counts in lungs. (b. c) Quantitative real-time PCR results for indicated gene expression normalized to GAPDH for total lung cells. (d. e) GSEA shows top enriched pathways between acute injury models and emphysema models.

Data shown are representative of three independent experiments. Data are mean ± s.e.m. ^*^*P*<0.05, ^**^*P*<0.01 and ^***^*P*<0.001. By two-tailed unpaired t-test. In b-c, n=6 mice per group.

**
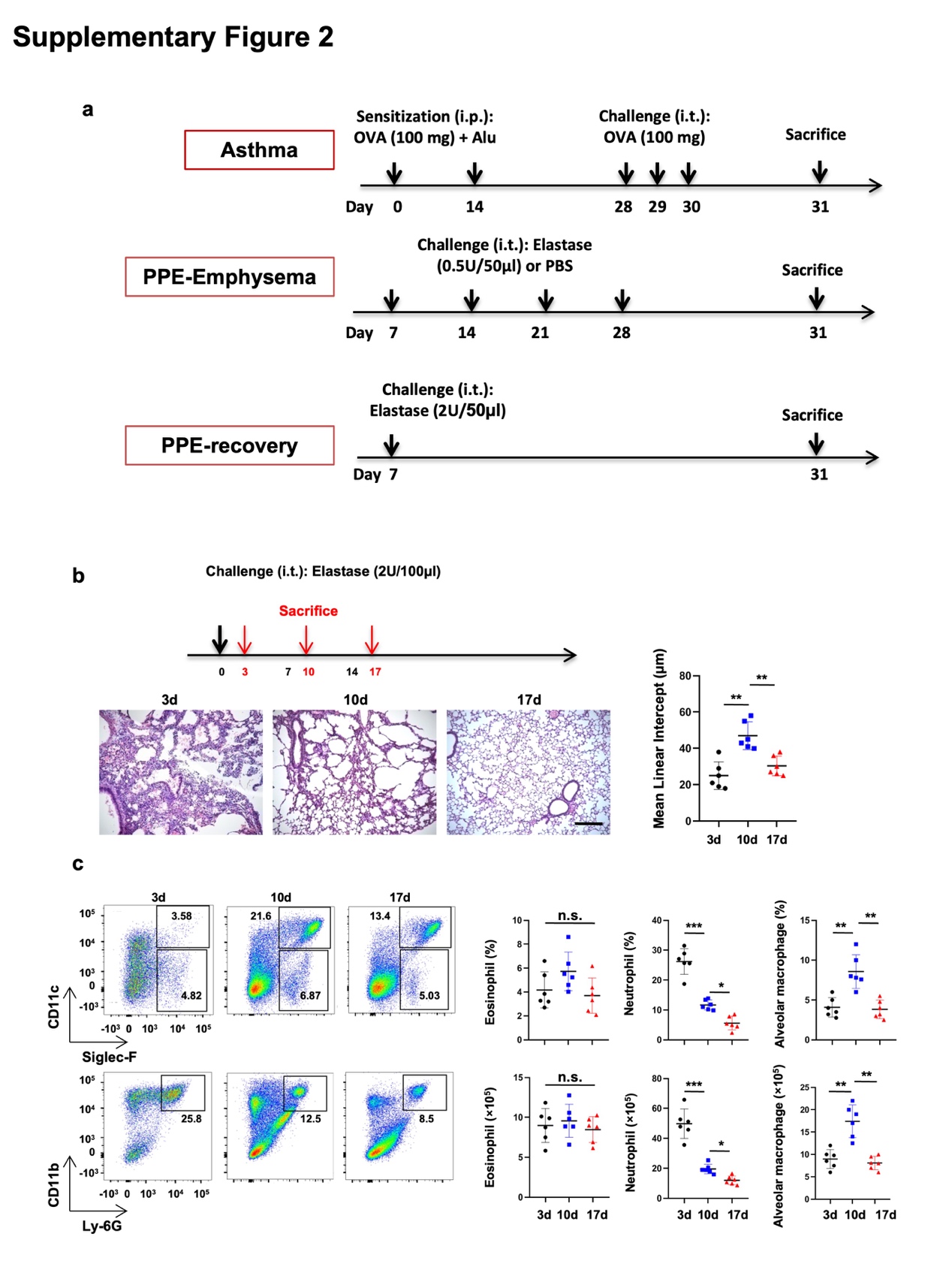
**

**
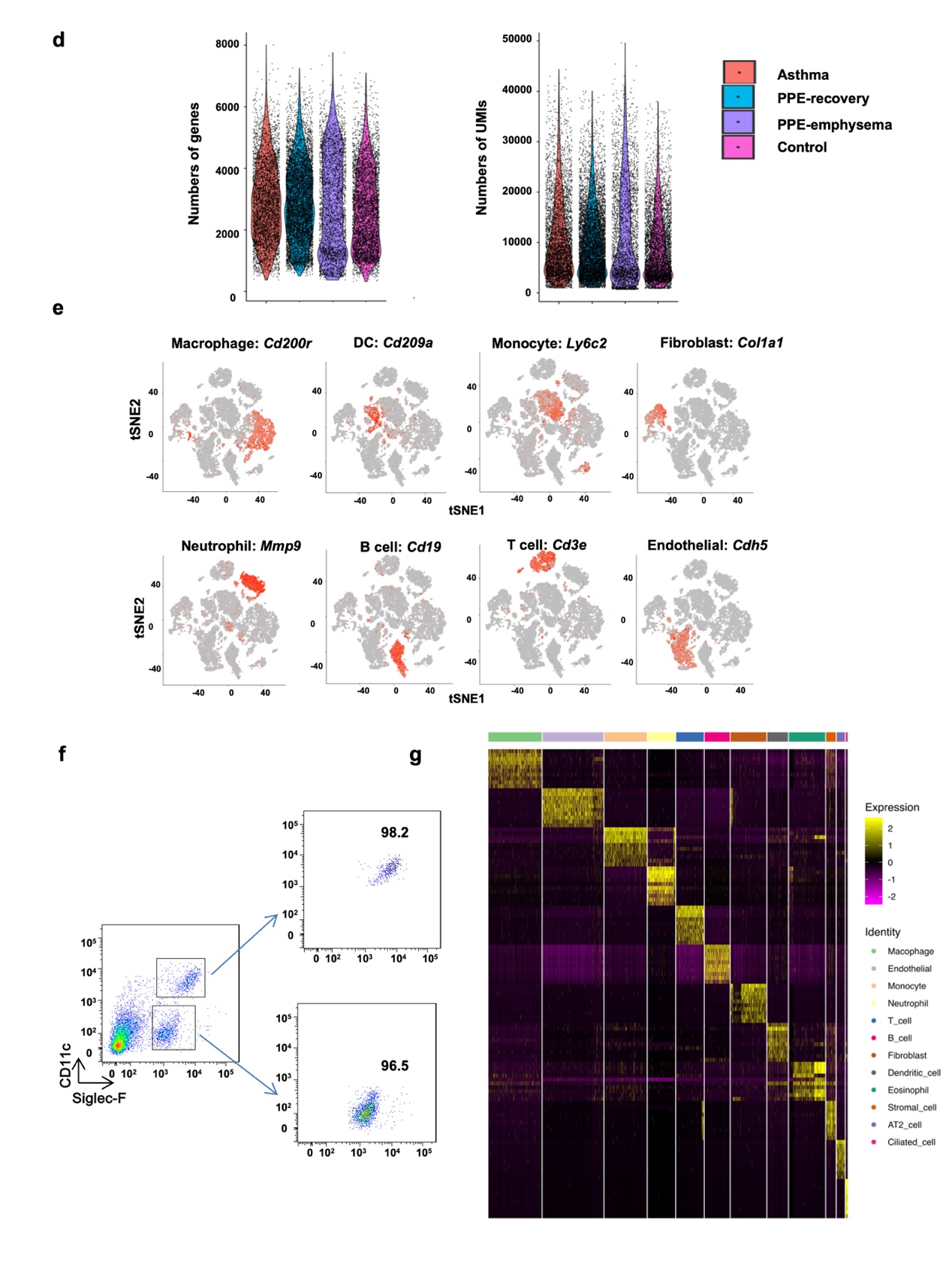
**

**Figure S2. Characterization of lung cell subpopulations.**

(a) Animal experimental protocol. Mice were randomly assigned into four groups: Asthma models, PPE-emphysema models, PPE-recovery models and control groups. In asthma models, WT mice were sensitized with OVA for two times, challenged with OVA for three times, then sacrificed for subsequent assay at day 31. In PPE-emphysema groups or control groups, animals received four intratracheal instillations at 1-week intervals and sacrificed three days after the last challenge. For PPE-recovery models, animals received only one intratracheal instillations at day 7 and were sacrificed at day 31. (b) In PPE-recovery models, mice were given only one challenge of PPE and sacrificed at indicated time. Pulmonary pathological sections were stained with hematoxylin/eosin (HE) (Bars represent 100 μm), mean linear intercept in the treated mice's lungs were measured. (c) Total lung cells were analyzed for eosinophils, neutrophils, and alveolar macrophages for the indicated treatment by flow cytometry. (d) Violin plots of the number of genes and number of UMIs of lung cells. (e) Expression of marker genes for major cell types. (f) Gating strategy for cell isolation by flow cytometry. (g) The heatmap of the top 10 significantly differentially expressed (SDE) genes in each cell type. In b-c, data shown are representative of three independent experiments. Data are mean ± s.e.m. ^*^*P*<0.05, ^**^*P*<0.01 and ^***^*P*<0.001. By two-tailed unpaired t-test. n=6 mice per group.

**
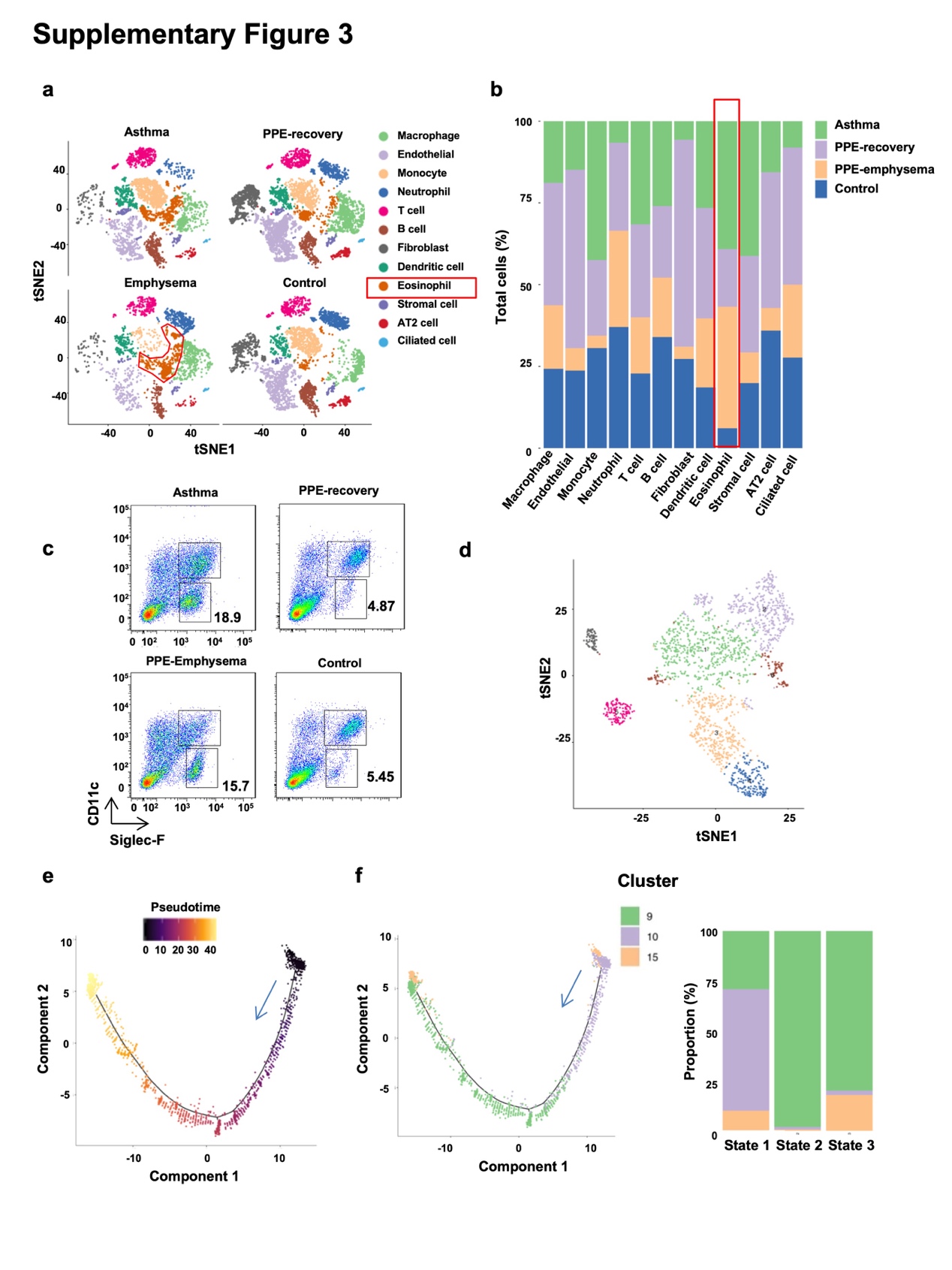
**

**Figure S3. Characterizing distinct states of eosinophils.**

(a) tSNE plot of single cells profiled in the presenting work colored by major cell types in indicate experimental models. (b) The proportion for each cell type in different experimental models. (c) FACS analyzing strategy of eosinophils. (d) t-SNE plot of seven eosinophil subclusters. (e) The developmental pseudotime of eosinophils inferred by analysis with Monocle 2. Color key from dark to bright indicates disease progression from early to late. (f) Cell state and proportions of eosinophils in Cluster 9,10,15 by analysis with Monocle 2.

**
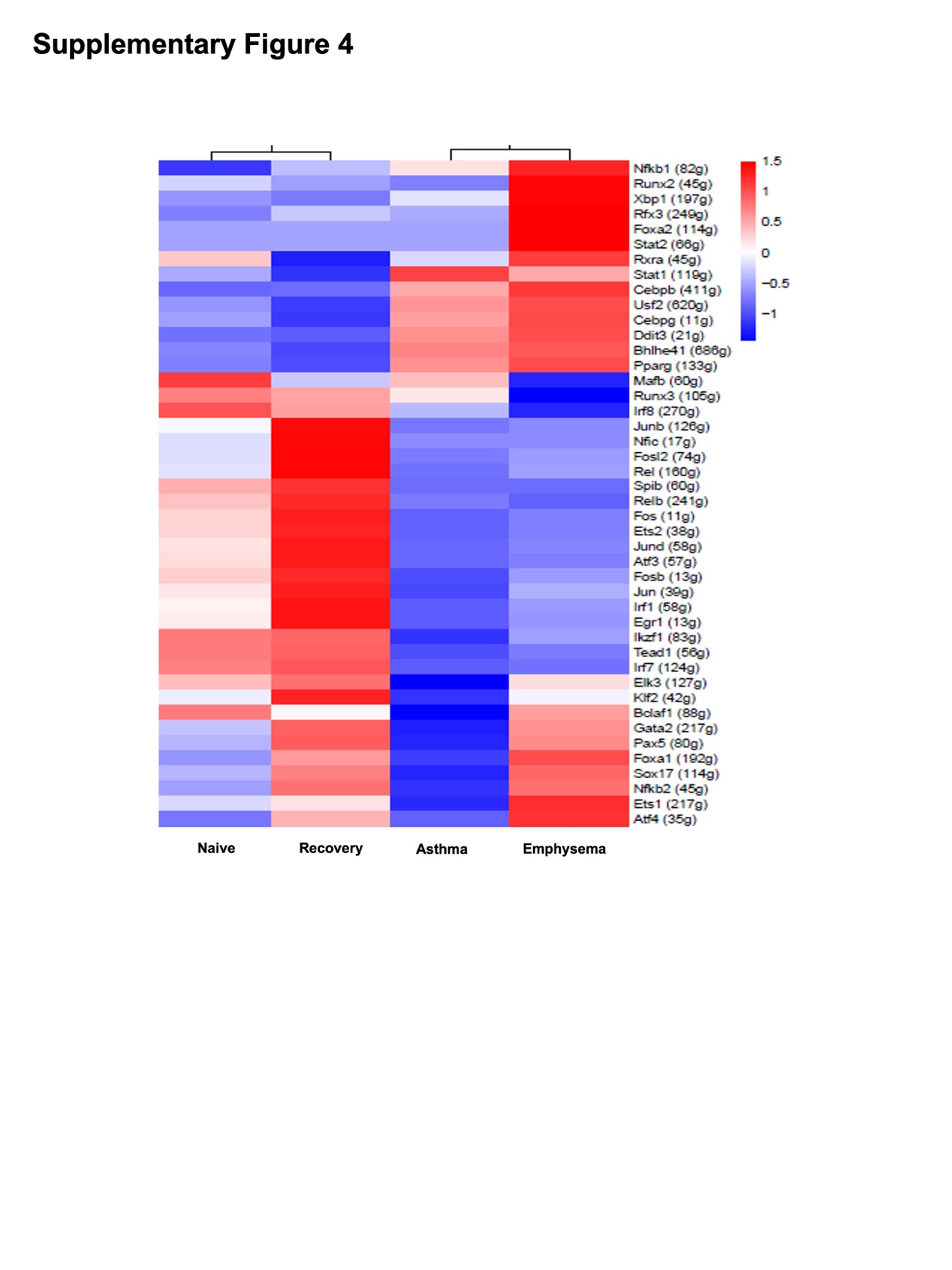
**

**Figure S4. Transcription factors drive eosinophil maturation and differentiation**.

Heat map shows the expression of transcription factors of eosinophils in each experiential model.

**
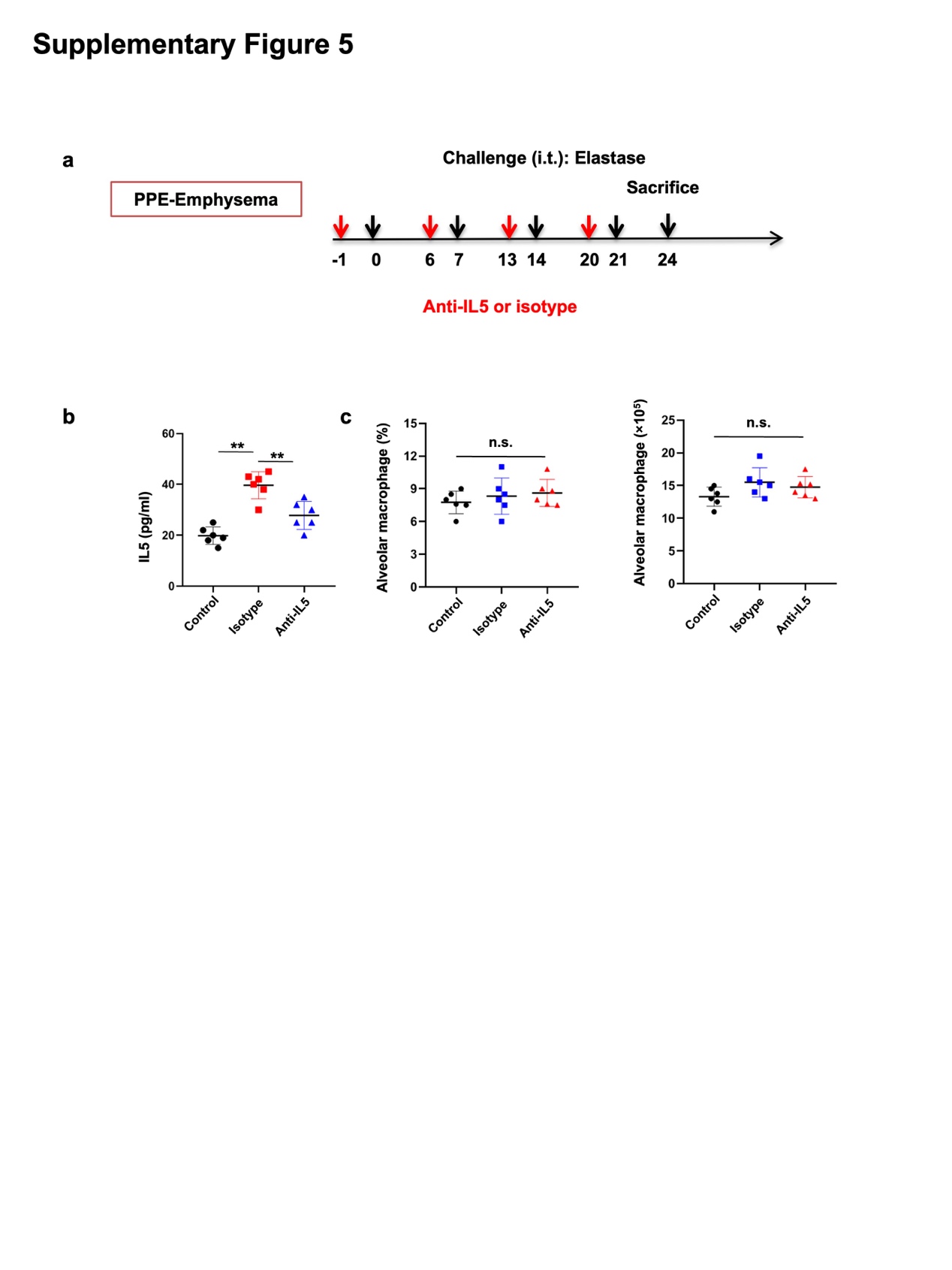
**

**Figure S5. Eosinophils are essential for emphysema development**.

(a**)** Mice were pretreated with 200 μg anti-IL-5 or control IgG per mouse or four times at 12 hours before the instillation of PPE, then scarified three days after the last PPE challenge. (b) ELISA of IL-5 in extracts of homogenized lungs. (c) Percentage and number of macrophages counts in lungs of PPE-treated mice, Data shown are representative of three independent experiments. The data are presented as the mean ± s.e.m. *P < 0.05, **P < 0.01 and ***P<0.001; two-tailed t-test. In a-c, n=6 mice per group.

**
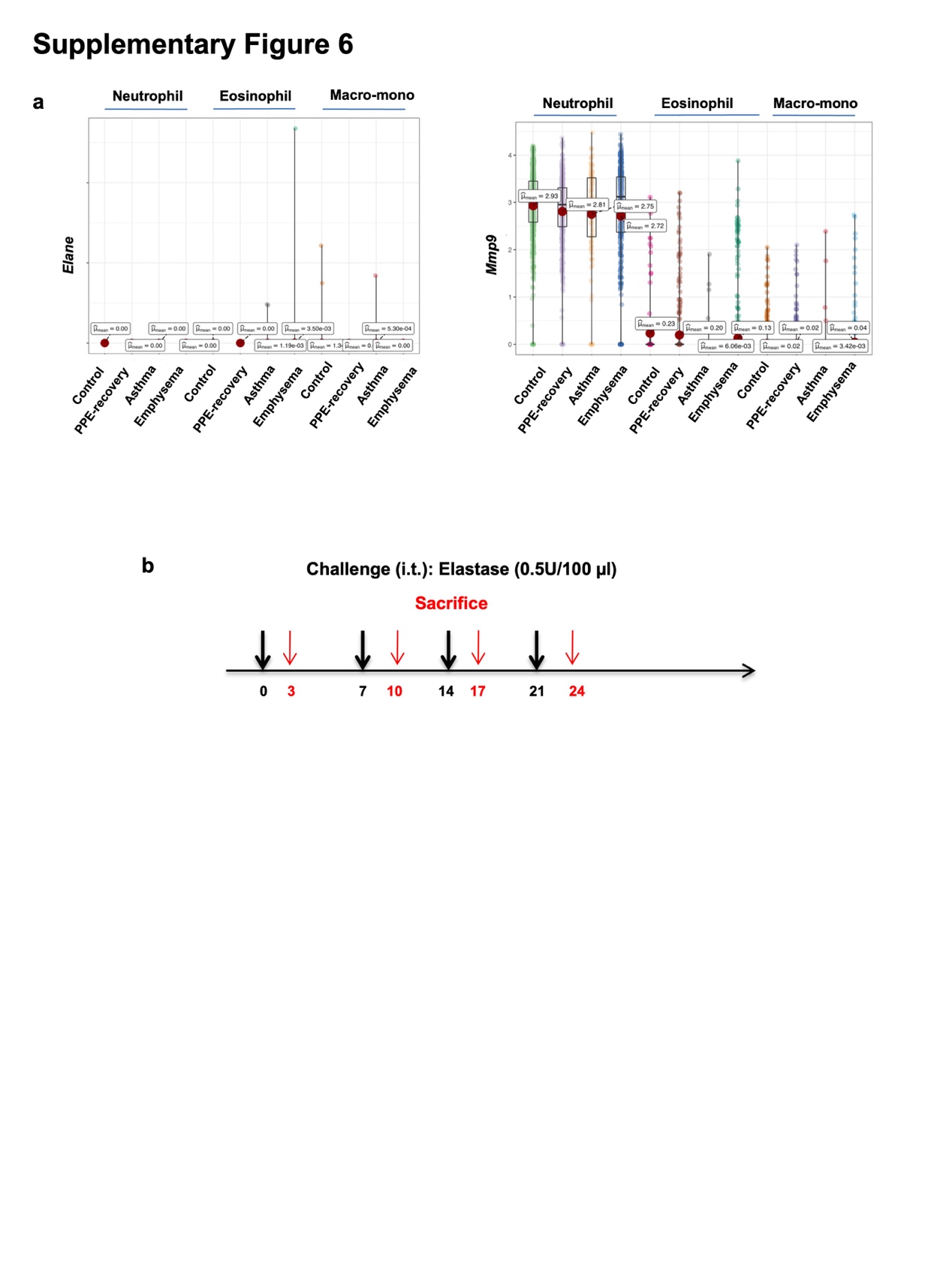
**

**Figure S6. Cathepsin L level increased in eosinophils of PPE-induced emphysema**.

(a) Expression of *Elane* and *Mmp9* in different cell types. (b) Animal experimental protocol. animals received four intratracheal instillations at 1-week intervals and sacrificed at indicate time for the following experiments.

**
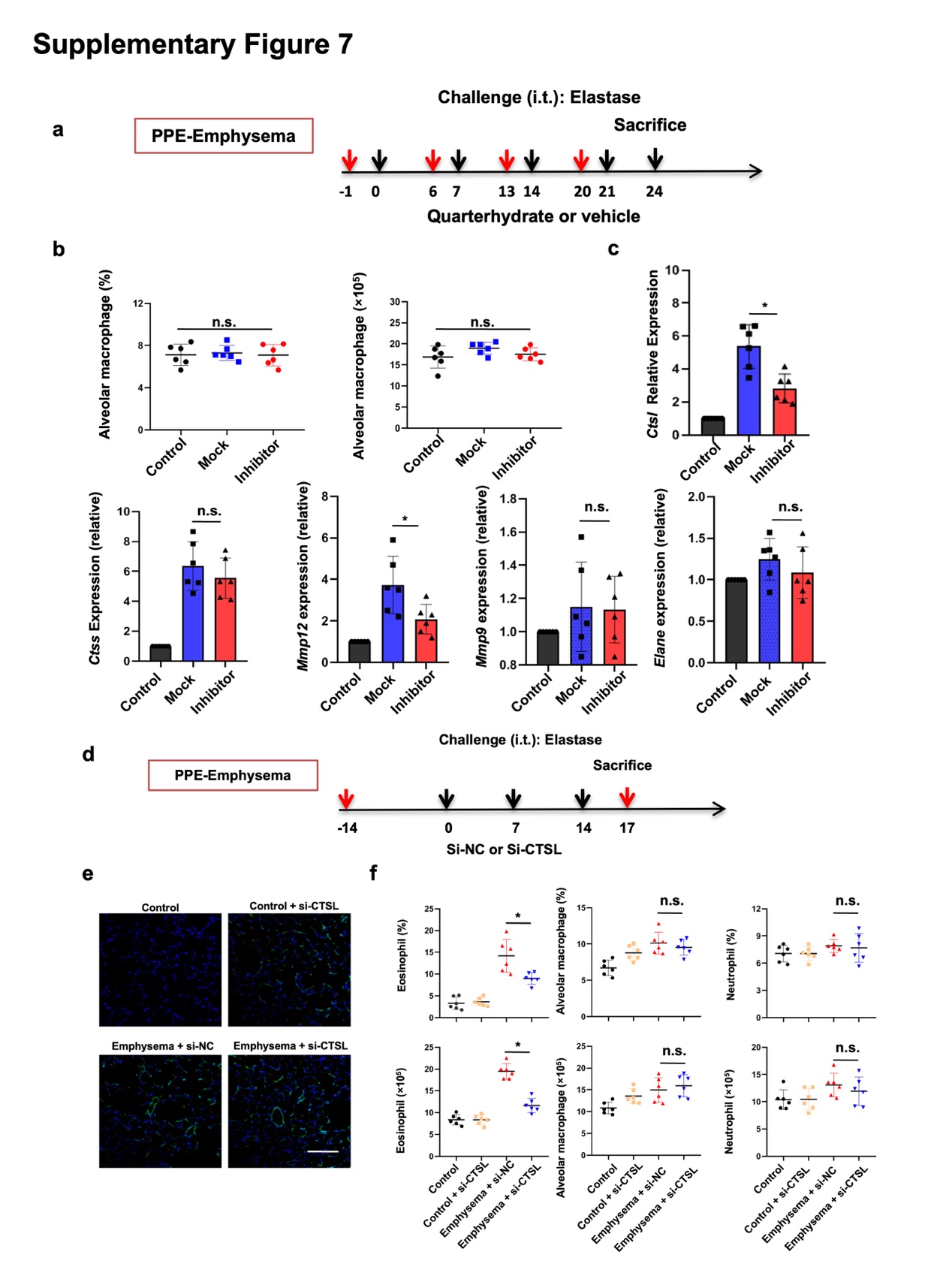
**

**Figure S7. Cathepsin L derived from eosinophils are crucial for emphysema**.

(a) Mice were pretreated with 500 μg CTSL inhibitor (SID 26681509, quarterhydrate) or vehicle control per mice for four times at 12 hours before the instillation of PPE, then scarified three days after the last challenge. (b) Flow cytometry analysis of alveolar macrophages in lungs. (c) Quantitative real-time PCR results for *Ctsl*, *Ctss*, *Mmp12, Mmp9* and *Elane* expression normalized to GAPDH for total lung cells. (d) Fourteen days before emphysema model construction, mice given AAV-m-CTSL (1.5×10^12^vg/ml) or control adeno-associated virus in 60 μl PBS via intratracheally ( i . t . ) administration to induce CTSL down-expression in the lung. Then animals received intratracheal instillations of PPE or PBS once a week for 3 weeks, at day 3 after the third challenge, mice were sacrificed for following experiment. (e) Fluorescence images of EGFP in lung tissues of control, control-si-CTSL, emphysema-si-NC and emphysema-si-CTSL group. Bar: 150µm. (f) Total lung cells were isolated and analyzed for eosinophil, neutrophil, and alveolar macrophage for the indicated treatment by flow cytometry. Data shown are representative of three independent experiments. ^*^*P*< 0.05, ^**^*P*< 0.01 and ^***^*P*<0.001; by two-tailed unpaired t-test. In a-f, n=6 mice per group.

**
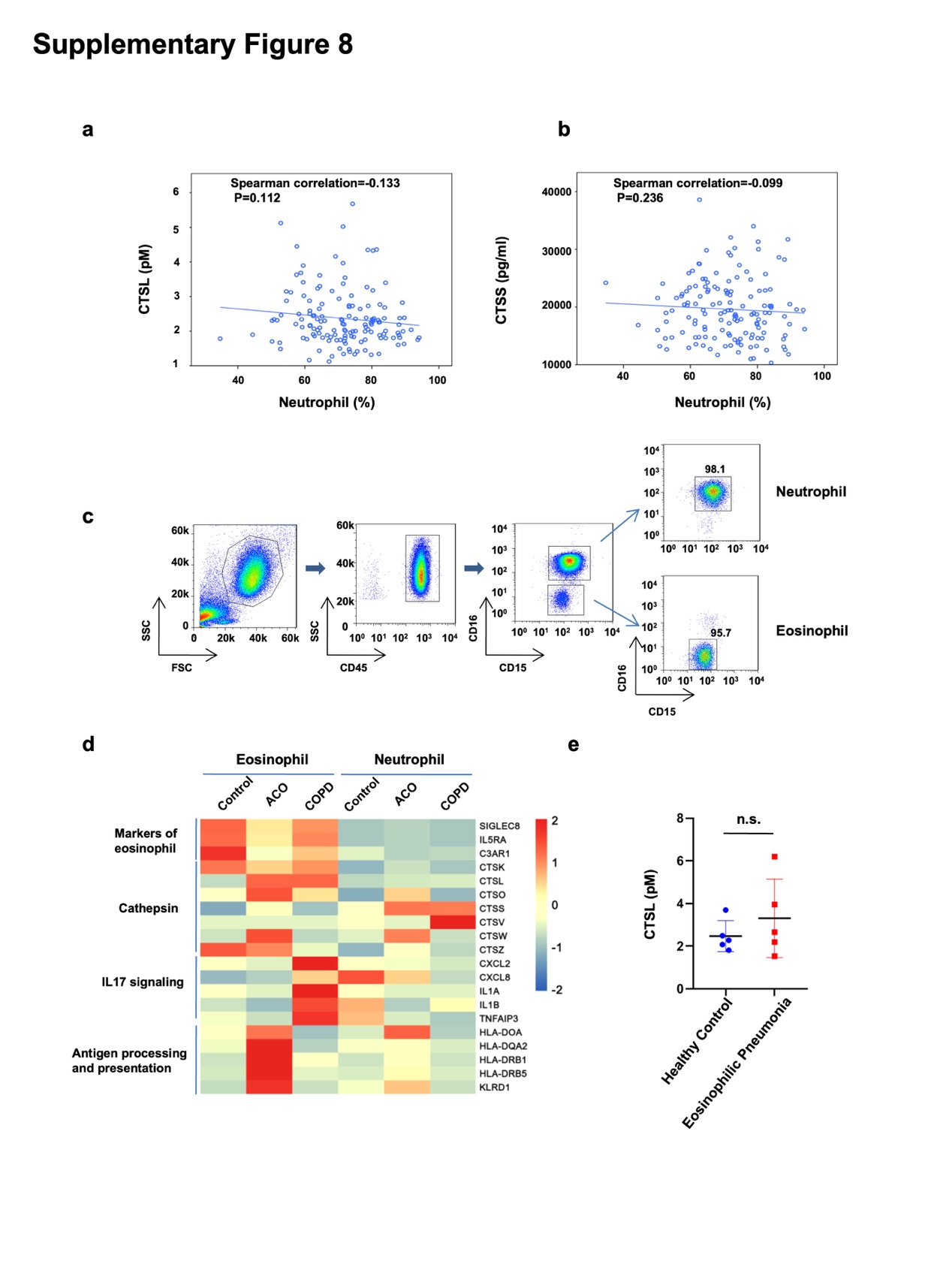
**

**Figure S8. The expression of CTSL in eosinophils is related to emphysema in COPD patients.**

(a, b) Spearman rank correlation analysis of the relationship between serum total CTSL，CTSS and neutrophil level in COPD patients. In a-b, n=144 for COPD patients. (c) PBMCs were removed from the whole blood cells, after RBC lysis buffer treatment, the cells were stained with corresponding antibodies and isolated by FACS. Gating strategy by flow cytometry for eosinophil (CD45^+^CD15^+^CD16^-^) and neutrophil (CD45^+^CD15^+^CD16^+^) counts. (d) Heat map shows the expression of indicated genes of eosinophils and neutrophils isolated from PBMCs. (e) Comparison of serum levels of CTSL between healthy controls or pneumonia patients by ELISA. In e, ^*^*P*< 0.05, ^**^*P*< 0.01 and ^***^*P*<0.001; by Mann–Whitney U-test. n=5 per group. ACO: asthma-COPD overlap.

**Supplementary Tables:**

**Supplementary Table 1. Basic information of COPD patients**

| **Variables *** | **Values (n =56)** |
| --- | --- |
| Age, years | 65 (58, 69) |
| Male, n (%) | 41 (73.2) |
| BMI, kg/m^2^ | 23.2 (21.1, 25.2) |
| Current smokers, n (%) | 23 (41.1) |
| Pack of years for smoking | 10 (0, 18) |
| ICS use, n (%) | 28 (50) |
| FEV_1_, L  FEV_1_%pred, % | 1.72±0.73  57.50±12.33 |
| GOLD I stage, n (%) | 21 (37.5) |
| GOLD II stage, n (%) | 25 (44.6) |
| GOLD III-IV stage, n (%) | 10 (17.9) |
| FVC, L | 3.31±0.96 |
| FEV_1_/FVC, % | 50.62±12.11 |
| Airway hyperresponsiveness, n (%) | 2 (3.6) |
| DLCO SB%pred | 53.4 (38.3, 68.7) |
| IgE, kU/L | 47.7 (30.7, 66.3) |
| Eosinophil, 10^9^/L | 0.17 (0.09, 0.25) |
| Eosinophil ≥ 0.2*10^9^/L, n (%) | 24 (42.9) |
| Neutrophil, 10^9^/L | 4.06±1.07 |
| Lymphocyte, 10^9^/L | 1.84±0.54 |
| Monocyte, 10^9^/L | 0.44±0.14 |
| Emphysema index (LAA%) | 10.2 (1.7, 17.1) |
| LAA%≥ 10%, n(%) | 29 (51.8) |
| PD15(g/L) | 91.07±31.25 |

Notes: * Continuous variables were expressed as mean ± SD or median (interquartile range) as appropriate. Categorical variables were n (%). Abbreviations: DLCO SB = Diffusing Capacity of the Lung for Carbon Monoxide measured by single breathe method; GOLD = Global Initiative for Chronic Obstructive Lung Disease; ICS = inhaled corticosteroids; PD15=15th percentile; LAA% = low lung attenuation area percent

**Supplementary Table 2. Multiva**riable logistic analysis for factors associated with emphysema in COPD patients

| **Risk factors** | **OR (95% CI)** | **P value** |
| --- | --- | --- |
| Eosinophil (10^9^/L) |  |  |
| Group 1 | reference |  |
| Group 2 | 46.82 (6.12-358.33) | <0.001 |
| Age, years | 1.03 (0.92-1.14) | 0.500 |
| Male, n (%) | 3.37 (0.35-53.62) | 0.243 |
| Pack of years for smoking | 1.13 (1.02-1.24) | 0.014 |
| ICS use, n (%) | 0.55 (0.07-4.50) | 0.632 |
| IgE, kU/L | 1.02 (1.00-1.04) | 0.054 |

Notes: Group 1, 0<=eosinophil<0.2; Group 2, Eosinophil ≥ 0.2, (ref: 0<=eosinophil<0.2).

Emphysema was defined as LAA% ≥10%.

Multivariable logistic regression was used for association of eosinophil with emphysema. Multi-collinearity was examined for independence of covariates

**Supplementary Table 3. Antibodies used in this study**

| **Antibody** | **Catalog number** | **Distributor** |
| --- | --- | --- |
| **For Flow cytometry** |  |  |
| PE-anti-mouse SiglecF (E50-2440) | 562068 | BD Pharmingen |
| PerCP-Cy5.5 -anti-mouse CD11b (M1/70) | 561114 | BD Pharmingen |
| APC-anti-mouse CD11c(HL3) | 561119 | BD Pharmingen |
| PerCP-Cy5.5 -anti-mouse CD45 (30-F11) | 550994 | BD Pharmingen |
| Flxable Viability Dye eFluor 780 | 65-0865-14 | eBioscience |
| FITC-anti-human CD45(HI30) | 555482 | BD Pharmingen |
| PE-anti-human CD15(HI98) | 555402 | BD Pharmingen |
| APC-anti-human CD16( B73.1) | 561304 | BD Pharmingen |
| **For Western blot** |  |  |
| Cathepsin L( CPL33/1) | C4618 | Sigma |
| β-Actin | AC026 | ABclonal |
| **For Immunofluorescence** |  |  |
| Ribonuclease A3 (RNASE3) | [MBS2090412](https://www.mybiosource.com/monoclonal-mouse-antibody/ribonuclease-a3-rnase3/2090412) | MyBioSource |
| ECP | orb156688 | Biorbyt |
| Prg2( BMK-13) | CBL419 | Sigma |
| Anti-Cathepsin L + V(33/2) | ab6314 | Abcam |
| DAPI | 10236276001 | Roche |
| **For Neutralization** |  |  |
| Purified NA/LE Rat Anti-Mouse/Anti-Human IL-5(TRFK5) | 554391 | BD Pharmingen |

**Supplementary Table 4.Primers and si-RNA sequences used in this study**

| **si-RNA sequences for adenovirus gene delivery** | |
| --- | --- |
| si-CTSLtarget sequence: | CAGAAGACTGTATGGCACGAA |
| si-RNA (Negative control) | TTCTCCGAACGTGTCACGTAA |
| **Primer sequences for qPCR** | |
| Mouse TNF-F | GATCGGTCCCCAAAGGGATG |
| Mouse TNF-R | CCACTTGGTGGTTTGTGAGTG |
| Mouse MMP9-F | CCGACTTTTGTGGTCTTCCC |
| Mouse MMP9-R | GCGGTACAAGTATGCCTCTGC |
| Mouse IL1β-F | GAAATGCCACCTTTTGACAGTG |
| Mouse IL1β-R | TGGATGCTCTCATCAGGACAG |
| Mouse IL6-F | TAGTCCTTCCTACCCCAATTTCC |
| Mouse IL6-R | TTGGTCCTTAGCCACTCCTTC |
| Mouse CXCL1-F | ACTGCACCCAAACCGAAGTC |
| Mouse CXCL1-R | TGGGGACACCTTTTAGCATCTT |
| Mouse S100A9-F | ATACTCTAGGAAGGAAGGACACC |
| Mouse S100A9-R | TCCATGATGTCATTTATGAGGGC |
| Mouse Atp6v0d2-F | CTGGTTCGAGGATGCAAAGC |
| Mouse Atp6v0d2-R | GTTGCCATAGTCCGTGGTCTG |
| Mouse CCL11-F | GAATCACCAACAACAGATGCAC |
| Mouse CCL11-R | ATCCTGGACCCACTTCTTCTT |
| Mouse CCL17-F | CAGGAAGTTGGTGAGCTGGT |
| Mouse CCL17-R | GACAGTCAGAAACACGATGGC |
| Mouse CTSS-F | CCACGCTGCCATCAGAAGAT |
| Mouse CTSS-R | TCCCAATGGTAGTCCAGGGT |
| Mouse CTSK-F | TAGCACCCTTAGTCTTCCGC |
| Mouse CTSK-R | CTTGAACACCCACATCCTGC |
| Mouse CTSL-F | ACAGAAGACTGTATGGCACGA |
| Mouse CTSL-R | GTATTCCCCGTTGTGTAGCTG |
| Mouse GAPDH-F | GAGAACTTTGGCATTGTGG |
| Mouse GAPDH-R | ATGCAGGGATGATGTTCTG |

**Supplementary original and uncropped films of Western blots**


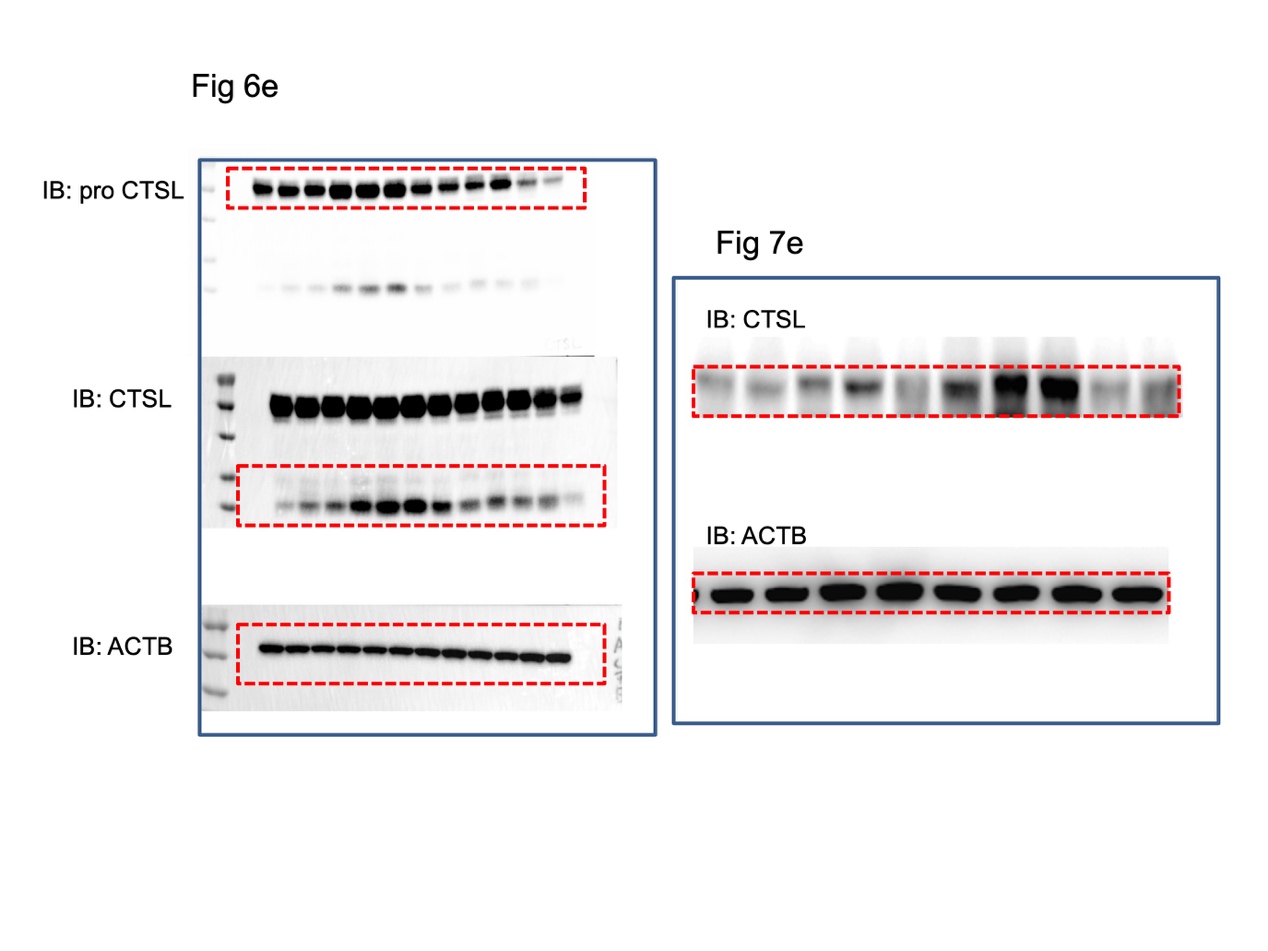

Supplement: Supplementary file 1 — Supplementary materials [file 41392_2023_1634_MOESM1_ESM.doc]
